# On the origin of life in the Zinc world. 2. Validation of the hypothesis on the photosynthesizing zinc sulfide edifices as cradles of life on Earth

**DOI:** 10.1186/1745-6150-4-27

**Published:** 2009-08-24

**Authors:** Armen Y Mulkidjanian, Michael Y Galperin

**Affiliations:** 1 School of Physics, Universität Osnabrück, D-49069 Osnabrück, Germany; 2 A.N. Belozersky Institute of Physico-Chemical Biology, Moscow State University, Moscow, 119991, Russia; 3 National Center for Biotechnology Information, National Library of Medicine, National Institutes of Health, Bethesda, MD 20894, USA

## Abstract

**Background:**

The accompanying article (A.Y. Mulkidjanian, *Biology Direct *4:26) puts forward a detailed hypothesis on the role of zinc sulfide (ZnS) in the origin of life on Earth. The hypothesis suggests that life emerged within compartmentalized, photosynthesizing ZnS formations of hydrothermal origin (the Zn world), assembled in sub-aerial settings on the surface of the primeval Earth.

**Results:**

If life started within photosynthesizing ZnS compartments, it should have been able to evolve under the conditions of elevated levels of Zn^2+ ^ions, byproducts of the ZnS-mediated photosynthesis. Therefore, the Zn world hypothesis leads to a set of testable predictions regarding the specific roles of Zn^2+ ^ions in modern organisms, particularly in RNA and protein structures related to the procession of RNA and the "evolutionarily old" cellular functions. We checked these predictions using publicly available data and obtained evidence suggesting that the development of the primeval life forms up to the stage of the Last Universal Common Ancestor proceeded in zinc-rich settings. Testing of the hypothesis has revealed the possible supportive role of manganese sulfide in the primeval photosynthesis. In addition, we demonstrate the explanatory power of the Zn world concept by elucidating several points that so far remained without acceptable rationalization. In particular, this concept implies a new scenario for the separation of Bacteria and Archaea and the origin of Eukarya.

**Conclusion:**

The ability of the Zn world hypothesis to generate non-trivial veritable predictions and explain previously obscure items gives credence to its key postulate that the development of the first life forms started within zinc-rich formations of hydrothermal origin and was driven by solar UV irradiation. This concept implies that the geochemical conditions conducive to the origin of life may have persisted only as long as the atmospheric CO_2 _pressure remained above ca. 10 bar. This work envisions the first Earth biotopes as photosynthesizing and habitable areas of porous ZnS and MnS precipitates around primeval hot springs. Further work will be needed to provide details on the life within these communities and to elucidate the primordial (bio)chemical reactions.

**Reviewers:**

This article was reviewed by Arcady Mushegian, Eugene Koonin, and Patrick Forterre. For the full reviews, please go to the Reviewers' reports section.

## Background

### Energetic aspects of the origin of life

The problem of origin of life on Earth (abiogenesis) remains one of the central and most intractable problems of modern biology. The current hypotheses cluster either around the "replication first" paradigm or the "metabolism first" concept, see [1-10] for consideration of the controversy between the two concepts. The "replication first" paradigm implies that formation of the first replicating entities preceded the origin of metabolism. This concept has grown from the so-called heterotrophic theory of abiogenesis that can be traced to Oparin, who had suggested that formation of complex proteinaceous complexes could proceed spontaneously under the conditions of reducing primordial atmosphere [11,12]. The Oparin's proposal, which was the first *detailed *scenario of abiogenesis, found an experimental confirmation. It has been shown later that simple building blocks, such as amino acids and carbohydrates, indeed, could build up from inorganic compounds under the conditions imitating the reduced primeval atmosphere, provided that external energy was delivered in the form of electric discharges or UV light [13-16]. The modern successors of Oparin's hypothesis are various RNA World scenarios, where the first RNA-like molecules are seen as capable both of self-reproduction and simple metabolism and thus preceding both proteins and DNA [17-33]. The "replication first" concept has been further supported by isolation and characterization of RNA enzymes (ribozymes) with different catalytic activities (see [27,29,31,34,35] and references therein). In addition, oligonucleotides of up to 30–50 units could be obtained in abiogenic systems from chemically activated monomers (e.g. nucleoside 5'-phosphorimidazolides [36]) when either polynucleotide chains [36-38] or mineral surfaces [39-44] were used as polymerization templates.

Still, the heterotrophic theory of abiogenesis has encountered certain problems. Oparin's initial model implied that primordial atmosphere was reducing, dominated by methane and hydrogen gas [11,12]. However, according to the current views, the primordial atmosphere was more oxidized and similar to those of modern Mars and Venus, where CO_2 _still makes 95% of the atmosphere with N_2 _and H_2 _being present in small amounts [45-53]. In a CO_2_-dominated atmosphere, any primordial (bio)chemistry could not start unless CO_2 _was reduced to organic molecules capable of participating in pre-biological syntheses (see [16] and references therein). Straightforward attempts to achieve abiogenic syntheses of amino acids or nucleobases with CO_2_-dominated gas mixtures so far proved unsuccessful [16,54].

The alternative "metabolism first" concept implies that emergence of the first replicators was preceded by establishment of self-sustaining cycles of chemical reactions that could produce increasingly complex organic compounds (see [10,55] for recent surveys). Currently, there are two detailed evolutionary scenarios representing the "metabolism first" concept. Wächtershäuser envisioned "two-dimensional" primordial metabolic cycles driven by oxidation of iron monosulfide (FeS) into iron disulfide (FeS_2_, pyrite) and confined to the mineral surfaces at the sea floor [56-61]. Besides the involvement of FeS clusters in a variety of anaerobic enzymes, consideration of FeS/FeS_2 _metabolism had an added benefit of accounting for the key role of sulfur in cell metabolism. Russell and co-workers [62-71], in turn, have suggested that the first metabolic cycles started inside porous chimneys of the deep-sea alkaline hydrothermal vents. It has been suggested that such compartmentalized structures consisting of FeS could offer three-dimensional reaction space and provide a framework for the emergence of the first cells [64].

From the viewpoint of energetics, any hypothesis of abiogenesis has to indicate explicitly the energy source(s) that could account for the (i) formation of reduced carbon compounds and (ii) primordial polymerization reactions. Therefore, it might be useful to compare the available scenarios of abiogenesis with respect to the underlying energy mechanisms. When considering the field from this point of view, it transpires that the proponents of the "replication first" scenarios, and in particular of the RNA World concept, just do not focus on primordial energetics and leave all options open (see e.g. [16]). Instead, more emphasis is put on understanding the chemistry of the primeval syntheses and the mechanisms of information processing in primordial replicating cycles [17-33].

**Figure 1:**
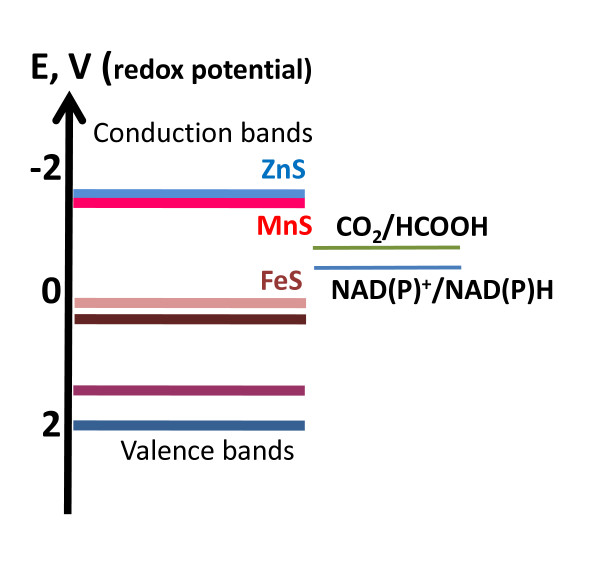
**Energy diagrams for FeS, ZnS, and MnS as potential donors of photo-excited electrons (left column) and for the biologically relevant electron acceptors (right column)**. The Highest Occupied Molecular Orbital (HOMO) level in the valence bands of each semiconductor is shown by a darker color than the respective Lowest Unoccupied Molecular Orbital (LUMO) level in the conduction band. The picture is based on data from references [72,99,122,262,264].

In contrast, proponents of the "metabolism first" concept explicitly address the energetics problem. Several papers by Wächtershäuser proposed a detailed chemical mechanism where oxidation of FeS to FeS_2 _at the sea floor was used to drive the reduction of either CO_2 _or CO [56-61]. Indeed, the free energy of the redox transition of FeS to FeS_2_, at least under some conditions, is sufficient to drive the reduction of CO_2_. Unfortunately, so far, all attempts to yield measurable CO_2 _reduction at the expense of FeS oxidation reaction under simulated "primordial" conditions have failed (see [72] and references therein). The reason for this failure, as discussed in detail by Schoonen and co-workers [72], is that for redox reactions, favorable thermodynamics alone is not sufficient. In addition, the redox potential of the electron donor has to be lower than that of the electron acceptor. To drive CO_2 _reduction at an appreciable rate, one needs a reducing agent with a redox potential that is lower than the redox potential of the CO_2_/formate redox pair, whereas the reducing potential of the FeS/FeS_2 _redox pair is higher than that (see Fig. 1). The reduction of CO by FeS is, in principle, possible and has been reported, albeit at unphysiologically high temperatures [59]. However, CO is not a major atmospheric constituent and probably never was one. In the atmospheres of Mars and Venus it is present in trace amounts. Instead, as argued by Schoonen and co-workers, it could arise in primordial settings predominantly *via *CO_2 _reduction; the reduction of CO_2 _to CO is, however, even less thermodynamically favorable than the reduction of CO_2_to formic acid [72]. Regarding the energetics of the primeval polymerization, Wächtershäuser speculated that ionic binding of the primordial building blocks to the FeS surfaces might facilitate their interaction and even make the synthetic reactions thermodynamically favorable [56-58].

In the most recent of the scenarios put forward by Russell and co-workers, hydrogen and hydrocarbons were produced below the sea floor in the complex "serpentinization" reactions and then brought to the surface by hydrothermal fluids (see [71] and references therein). Considering the primeval polymerization reactions inside the porous, compartmentalized bodies of hydrothermal vents, these authors suggested that the pH gradient across the inorganic membranes of these compartments, between the alkaline hydrothermal fluids and the more acidic primordial ocean, could have served as the energy source for primeval syntheses [70,71], by analogy with the transmembrane proton gradients on the membranes of modern bacterial cells. However, coupling of the transmembrane proton gradient to synthetic reactions – even in most primitive bacteria – is performed by a sophisticated enzyme machinery which seems to be evolutionarily recent [73]. Therefore it remains unclear whether and how such coupling could have occurred in the inorganic systems.

### The concept of the photosynthetic origin of life in the Zinc world

A few scholars have invoked the solar radiation as a potential source of energy for the origin of life (see e.g. [74-85]). However, the idea of driving the abiogenesis directly by solar energy has not won much support, despite the Sun being by far the most powerful energy source on this planet [16,45,86]. The limited acceptance of this idea is probably due to several factors. First, only short-length UV quanta carry enough energy to drive primeval organic syntheses in the absence of enzymes [75]. These quanta were available on the primordial Earth: in the absence of the ozone shield, the UV component of the solar light was by 2–3 orders of magnitude stronger than now [87,88]. However, the same UV quanta would cause photo-dissociation of organic compounds. Therefore, most scholars considered the UV irradiation of the primordial Sun to be a hazard for the first life forms and suggested searching for life origins at the sea floor (see e.g. [87]). Second, as the energy of UV quanta could be utilized for the synthetic reactions in many different ways, there has been no consensus on the particular mechanisms involved. For example, considering the ways of CO_2 _fixation, Mauzerall and co-workers advocated the idea of CO_2 _photo-reduction to formaldehyde in the presence of dissolved ferrous hydroxide [83,89,90], while other authors have argued that several naturally occurring minerals possess the properties of broad-band semiconductors and could perform abiogenic photosynthesis [81,91-93]. Further, since there are several minerals with this capacity, different authors advocated participation of different minerals in the primordial (photo)syntheses. Bard and co-workers [92,93], and, more recently, Senanayake and Idriss [94] studied the photosynthesis on the surface of TiO_2 _(anatase/rutile), Halmann and co-workers tested the CO_2 _photo-reduction by diverse minerals getting a high outcome with WO_3 _(wolframite) [81], while Schoonen and co-workers investigated MnS (alabandite [95]) and ZnS (sphalerite [96]). Last but not least, no detailed – and hence testable – scenario of light-driven abiogenesis has been suggested so far.

The first such detailed scenario has been put forward in the accompanying article [97]. This scenario centers on the role of zinc sulfide (ZnS) as a compound that uniquely combines several traits that could be decisive for the emergence of life on Earth. Among others, the arguments included ZnS crystals being, on the one hand, extremely efficient photo-catalysts capable of reducing CO_2 _and other organic compounds with a quantum yield of up to 80% [96,98-103] and, on the other hand, common constituents of the hydrothermal vent systems typically coating those that eject fluids with the temperature in the 200 – 300°C range [104-106]. The Zinc world hypothesis suggests that, as long as the atmospheric pressure at the surface of primordial Earth remained above ca. 10 bar, porous ZnS formations could build up in the direct reach of UV-rich sun beams, because the temperature of liquid water in hot springs could remain above 200°C even in sub-aerial settings. In this "Zn world", the energy of light could be used (i) for the abiogenic, ZnS-mediated photosynthesis of diverse organic compounds, (ii) for the selection of most photochemically stable of them, and (iii) for driving the surface-catalyzed polymerization reactions (see [97] for details). This ZnS-mediated photosynthesis, however, would gradually decline with the drop in atmospheric pressure below 10 bar, with the ZnS-coated surfaces persisting only around deep-sea hydrothermal vents, which continue to extrude hot, Zn-rich fluids up to these days. The hypothesis further envisions that after the submergence of the Zn-rich formations, sub-aerial biotopes had to cope with the impact of the generally abundant Fe^2+ ^ions. Iron, unlike zinc, is redox-active, so that the first life forms had to undergo major changes to adjust to the iron-containing, redox-active environment.

### Approaches to validation of evolutionary hypotheses

Obviously, experimental verification of this (or any other) evolutionary scenario would prove extremely difficult, if not impossible. However, as noted by Wächtershäuser [107], validation of such concepts could be accomplished through rigorous application of Karl Popper's principles of testing scientific theories [108,109]. These tests include (i) consideration of the experimental evidence, (ii) the relation of the hypothesis to earlier theories in the field, (iii) falsification tests, and (iv) demonstration of the explanatory power of the hypothesis. Popper described the falsification tests as follows: "*The purpose of this last kind of test is to find out how far the new consequences of the theory *– *whatever may be new in what it asserts *– *stand up to the demands of practice, whether raised by purely scientific experiments, or by practical technological applications. Here too the procedure of testing turns out to be deductive. With the help of other statements, previously accepted, certain singular statements – which we may call 'predictions' – are deduced from the theory; especially predictions that are easily testable or applicable. From among these statements, those are selected which are not derivable from the current theory, and more especially those which the current theory contradicts. Next we seek a decision as regards these (and other) derived statements by comparing them with the results of practical applications and experiments. If this decision is positive, that is, if the singular conclusions turn out to be acceptable, or verified, then the theory has, for the time being, passed its test: we have found no reason to discard it. But if the decision is negative, or in other words, if the conclusions have been falsified, then their falsification also falsifies the theory from which they were logically deduced*" (quoted from ref. [109]). As noted by Yarus and colleagues [25], falsification tests are especially important when hypotheses cannot be experimentally proven in principle, as in the case of evolutionary scenarios. Several falsification tests are then required, since multiple confirmed predictions would enhance the plausibility of the initial idea [25].

In the accompanying article [97], the available evidence for the Zn world hypothesis was considered, the underlying physical and chemical mechanisms were analyzed, and the suggested scenario was compared to other concepts of the origin of life. Here, in continuation of this analysis, we formulate and check a set of predictions that follow from the Zn world hypothesis. We also address the explanatory power of the hypothesis and discuss how the suggested concept could eventually be experimentally tested.

## Results

### Falsification tests of the Zinc world concept

As noted by Popper, predictions stemming from the tested hypothesis must be logically uncoupled from the premises on which the hypothesis had been based [109]. The Zn world hypothesis is based on the three key premises, namely (i) the experimentally demonstrated ability of ZnS crystals and nanoparticles to photoreduce CO_2 _with a high quantum yield [96,99-103,110], (ii) the need of metal-rich hydrothermal settings for the emergence of first organisms [45,47,62,63,65-67,111-114] and the frequent coating of such settings by ZnS [104,115-117], and (iii) the unique photostablity of polynucleotides that could imply their emergence in the presence of UV light as a selective factor [33,78,118-121]. Since all these premises are either physico-chemical or geological, it seemed worthwhile to focus here on the biological predictions stemming from this hypothesis. A specific trait of the Zn world would be the constantly elevated concentrations of Zn^2+ ^ions. Indeed, upon reduction of CO_2 _by photo-excited electrons, the negative charge of these electrons is compensated by protons coming from the water [99,122], according to the equation:

(1)

where ZnS* is the photo-excited state of a ZnS crystal, see Fig. 1. The resulting accumulation of positive charges in the illuminated ZnS crystals leads to their disruption and to the release of Zn^2+ ^ions, i.e. photo-corrosion. Photo-corrosion cannot be completely prevented even by applying efficient electron donors (so-called hole scavengers, see the accompanying article [97] for details and references). It remains the main obstacle in the practical applications of ZnS in photoelectric devices, so that the less photo-corrosive TiO_2 _is routinely used [122,123]. A similar photo-corrosion should have taken place within the primeval illuminated ZnS compartments and caused their enrichment in Zn^2+ ^ions. An environment with continuously elevated Zn^2+ ^content is geochemically unusual. Generally, Zn^2+ ^ions form poorly soluble salts with such widespread anions as phosphate, carbonate and sulfide, which is why the concentration of free Zn^2+ ^in modern seawater is less than 2 nM [104]. The concentration of Zn^2+ ^ions in primordial anoxic waters should have been lower than now because of the poor solubility of ZnS, the predominant Zn^2+ ^source in the ancient ocean [124]. The available estimates of Zn^2+ ^content in primordial waters are in the range of 10^-15^-10^-12 ^M [124-126].

Hence, if we could find evidence of the emergence of life in Zn^2+^-rich habitats, the Zn world hypothesis could be considered confirmed in its key postulate. Indeed, because the Zn^2+ ^ions should be continuously removed by precipitation, an elevated Zn^2+ ^content would be expected to persist only at the continuously photosynthesizing ZnS surfaces, i.e. both the ZnS surfaces and photosynthesis (as a sink for electrons) are required. Thus, any evidence of the origin of life in a primeval Zn^2+^-rich milieu is, at the same time, evidence of ZnS-mediated abiogenic photosynthesis at the primordial Earth. Such evidence could be obtained by examining properties of modern prokaryotic and eukaryotic cells: those RNA and protein molecules that stem from the Zn world could retain certain traits from their emergence in Zn^2+^-rich settings owing to the principle of chemistry conservation. As discussed in more detail elsewhere [8], this principle, which is implicitly acknowledged by natural scientists, entails that the organismal chemistry can retain information about ancient environmental conditions (see e.g. [45]). Apparently, post-modification of metabolic pathways in response to environmental changes is often either not possible or evolutionarily less probable than simply maintaining the "ancient" intrinsic chemical milieu. For example, the cell cytoplasm is highly reduced even in those organisms that inhabit oxygenated environments. The reduced state of the cytoplasm indicates that the first cells have evolved – and the principal biochemical pathways have been established – before the atmosphere became oxygenated (which was due to the activity of cyanobacteria at 2 – 2.5 Ga [127]). The principle of chemistry conservation can be applied to reconstruction of primordial environmental conditions even in those cases when no reliable geological evidence is available.

The Zn world concept suggests that the environment that housed the first life forms was enriched in Zn^2+ ^ions. Then the Zn^2+ ^ions, released upon photosynthesis, could interact with the polymers at the ZnS surface. The latter interaction could be thermodynamically favorable since the polymer molecule could potentially provide several coordinating bonds for a Zn^2+ ^ion (which can form up to 6 of them [128]). However, to use all these bonding modalities, the Zn^2+ ^ion had either to induce folding of the polymer around itself (see [129]) or to bind several polymer molecules together. Those RNA and protein molecules that succeeded in trapping Zn^2+ ^ions, in turn, could get selective advantage either as more stable or as catalytically active ones. These Zn-containing polymers would then be likely preserved in the course of evolution and could show up in the modern cells.

Based on these arguments, we have made a set of specific predictions related to the occurrence of Zn in modern organisms. In the subsequent sections, we test these predictions one by one. In order to avoid potential biases, we relied, wherever possible, on data extracted from the published literature and the publicly available databases. This testing turned out to be fairly complicated. Zn^2+ ^ions are spectroscopically elusive: unlike other biologically-relevant transition metals, such as Fe, Cu, and Mn, zinc has no characteristic spectral signatures either in optical (UV-visible) or in EPR spectra (see [130] and references therein). Therefore, analysis of the Zn content of biopolymers cannot rely on spectroscopy, which is, generally, the method of choice in bioinorganic chemistry. Instead, presence of a bound Zn^2+ ^ion in a biopolymer has to be revealed either by methods of analytical chemistry (see e.g. [131,132]), or from structural data, or from functional measurements, e.g. of the catalytic activity in the presence of different metal ions (see [133,134] and references therein), or by bioinformatics approaches [135].

We would like to emphasize that the Zn world concept contrasts a variety of models that center around the role of iron in the emergence of life, either as Fe^2+ ^ions in solution [77,83,89,90,136], or as iron sulfide [56-58,60-64,67,68,71,137,138]. Therefore, while analyzing the data, we specifically looked at the content of iron and other transition metals that are essential for life (hereafter "essential metals" [128]).

#### The presence of Zn^2+ ^in modern RNAs

The Zn world scenario implies that the first RNA-like oligomers emerged within the illuminated ZnS compartments. Then the Zn^2+ ^ions might be preserved in modern RNA molecules either as structural elements or catalytic cofactors.

##### Occurrence of Zn the RNA structures

The idea of modern RNA structures retaining the Zn^2+ ^ions that were trapped by the first RNA molecules emerging in the zinc-rich habitats leads to the prediction no. 1: *Known RNA structures should be enriched with Zn*^2+ ^*as compared to other transition metals*. To check this, we have turned to the solved RNA-containing structures, available in the Protein Data Bank (PDB, [139,140]) and Nucleic Acid Database [141,142].

A comparison of the transition metal content in the available RNA-containing structures, as listed in the MERNA (Metals in RNA) database [143,144], is presented in Table 1 (these data represent transition metal atoms located at the distance of no more than 6 Å from any atom that is part of the RNA molecule). RNA-containing structures from the PDB include such essential metals as Zn, Mn and Co, with Zn seen in 64 structures, more than any other transition metal. The relatively large number of Co-containing structures is due to the routine use of cobalt (III) hexamine as a standard stabilizing reagent which mimics hydrated magnesium [145]. Manganese atoms are seen in 16 structures, whereas no Fe atoms in the vicinity of RNA molecules have been reported. Our own further analysis, which used a shorter cut-off of 3 Å, has shown that the majority of these metal atoms interact not with RNA proper but with the side chains of various RNA-bound proteins. Still, in some cases we could find transition metal atoms that interacted directly with nucleotides, namely Zn in the PDB entries 1NLC, 1S03, 1YXP, 1D9F, Mn in the PDB entries 1EHZ, 1N35, 1Y3O, 2G81, and so on. The certain scarcity of these interactions is due to the poor binding of metal cations to RNA and, accordingly, the poor selectivity of such binding. Therefore transition metal atoms are usually seen in those structures that were crystallized from solutions that contained the respective salts. In the absence of added Zn or Mn salts, Mg atoms, which are present in standard crystallization media, bind in the respective positions (as could be judged from the comparative analysis of the RNA structures that were crystallized several times, with different divalent cations (see e.g. [146]). Thus, a separate question that, generally, deserves clarification is the nature of the divalent metal atoms that are bound by the RNA-protein complexes *in vivo*. We have tackled this question while trying to clarify the origin of Cd atoms in the RNA-containing structures. The presence of Cd in 42 RNA-containing structures, as reported in the MERNA database (Table 1), was intriguing since Cd is not an essential metal. We have checked the Cd-containing structures and found out that 41 of them represent different ribosomal structures which were crystallized in the presence of CdCl_2 _[147,148]; CdCl_2 _was apparently used to improve the crystal stability (Dr. Gulnara Yusupova, personal communication). The remaining Cd-containing structure shows a complex of the hammerhead ribozyme with substrate RNA that was crystallized in the presence of 25 mM of CdSO_4 _[149]. For the 41 structures related to the large ribosomal subunit, we could tentatively infer the nature of the metal ions that were replaced by the added Cd^2+ ^ions. Ramakrishnan and co-workers have recently crystallized the whole bacterial ribosome with bound tRNA and mRNA under physiological conditions, with Mg^2+ ^as the only divalent ion used upon preparation [150]. This structure contains dozens of Mg^2+ ^ions, a few Zn^2+ ^ions, and no Cd^2+ ^ions. Apparently the Cd^2+ ^ions in the 41 ribosomal structures occupied the loci that are normally occupied by either Mg^2+ ^or Zn^2+ ^ions. Since Ramakrishnan and co-workers crystallized the ribosome without adding Zn salts to the crystallization medium [150], the Zn atoms seen in that structure should be the retained native ones. These data show that, indeed, Zn atoms are found in RNA molecules and RNA-protein complexes much more often than any other transition metal atoms.

**Table 1:** Transition metal content in RNA structures.

Group	3 (IIIB)	4 (IVB)	5 (VB)	6 (VIB)	7 (VIIB)	8 (VIIIB)	9 (VIIIB)	10 (VIIIB)	11 (IB)	12 (IIB)
Period 4	Sc 0	Ti 0	V 2	Cr 0	**Mn** **16**	**Fe** **0**	**Co** **32**	**Ni** **0**	**Cu** **0**	**Zn** **64**

Period 5	Y 0	Zr 0	Nb 0	**Mo** **0**	Tc 0	Ru 1	Rh 4	Pd 0	Ag 0	Cd 42

Period 6	La 0	Hf 0	Ta 0	**W** **6**	Re 0	Os 3	Ir 3	Pt 1	Au 0	Hg 3

Essential metals are shown in bold font. The data are taken from the February 2007 release of the MERNA database [143,144], and reflect data from 389 individual RNA-containing structures. Each value represents the number of structures where the indicated transition metal atoms are located within 6 Å from the nearest RNA atom.

##### Role of Zn in the RNA catalysis

The metal ions that were trapped by primeval RNA polymers could serve as catalytic centers. This consideration leads to the prediction no. 2: *There should be ribozymes with Zn-dependent catalytic activities*.

The detailed catalytic mechanisms of natural ribozymes have been studied only in few cases (see [35,151-155] for recent comprehensive reviews). RNA molecules are surrounded by a shell of diverse cations that stabilize the negative charges of the backbone phosphate groups. Generally, the metal specificity of ribozymes is low, because of weaker, as compared to proteins, cation binding, so Mg^2+ ^ions, which are always present in large amounts as stabilizers, can seemingly occupy any metal-binding site. Therefore, the exact chemical nature of the catalytically-relevant metal ions is often difficult to determine. In most cases, the catalytic activity of ribozymes could be restored by several divalent cations, e.g. by Mg^2+^, Zn^2+^, Mn^2+^, Cd^2+^, Pb^2+^. In some cases, however, only particular ions, e.g. Zn^2+ ^[156,157] or Mn^2+ ^[158,159] were found to be functional. Taking into account that Cd and Pb do not belong to the essential metals [128], the available data (see e.g. [35,151-155,160-167]) indicate that the transition metals that can be relevant for natural RNA catalysis are, in the first line, Zn and Mn.

Summarizing the RNA-related predictions, it is worth noting that Fe has not been reported either as a structural constituent of RNA molecules or as a metal that is important for the RNA catalysis. The striking absence of iron atoms in the vicinity of natural RNA molecules is likely to be due to the danger of the RNA cleavage by hydroxyl radicals that could be produced in the presence of redox-active Fe^2+^/Fe^3+ ^ions [168-170]. Accordingly, it seems reasonable to suggest that early evolution of RNA proceeded in habitats that were enriched in Mg, Zn and Mn, but not in iron.

#### Traces of the Zn^2+^-rich environment in the evolutionarily old proteins

##### Occurrence of Zn the oldest protein folds

The same logics as used above to suggest the possibility of stabilization of the early RNA folds by photosynthetically released Zn^2+ ^ions is applicable to the first proteins, which, owing to the more versatile chemistry, could bind metal ions even tighter than RNA molecules do [128]. Zn^2+^-mediated protein folding has indeed been reported, see e.g. [171,172]. Hence, it is possible to formulate the prediction no. 3: *Zn*^2+ ^*ions should be associated with the evolutionarily oldest protein folds*.

Yang and colleagues recently checked the distribution of fold superfamilies (FSF) among 174 complete genomes [173]. Of 1294 FSFs only 49 were reported to be present in all Archaea, Bacteria and Eukarya with known complete genomes. It seems reasonable to assume that these FSFs are among the evolutionarily oldest ones. Table 2 contains data on metals associated with these 49 FSFs [174]. Because of certain elusiveness of zinc (see above), we have checked the metal content in those representatives of these 49 FSF whose structures could be found in the PDB. Zinc atoms were found in representatives of 37 FSFs. Cadmium atoms were present in the representatives of 21 FSFs. Again, since Cd is not an essential metal [128], Cd atoms most likely occupy the sites where Mg^2+ ^or Zn^2+ ^ions are normally bound (see above and [175]). Iron atoms were associated only with representatives of three FSFs. Manganese has been found in representatives of 19 FSFs, whereas Mg atoms were present in all FSFs except for one. These data support the idea that formation and stabilization of the oldest protein folds have occurred in the settings that were rich in Mg, Zn and Mn, but not in Fe.

**Table 2:** Occurrence of metal atoms in the representatives of the 49 fold superfamilies that are common to Bacteria, Archaea and Eukarya

**Name**	**Zn**	**Fe**	**Cd**	**Ni**	**Cu**	**Mn**	**Mg**
S13-like H2TH domain	3 (38)	0	0	0	0	0	3 (18)

S15/NS1 RNA-binding domain	1 (18)	0	0	0	0	0	1 (17)

Anticodon-binding domain of a subclass of class I aminoacyl-tRNA synthases	5 (21)	0	0	0	0	0	1 (2)

Ribosomal protein L11, C-terminal domain	0	0	0	0	0	0	1 (4)

5' to 3' exonuclease, C-terminal subdomain	2 (3)	0	0	0	0	2 (2)	2 (2)

Ribosomal protein S7	1 (18)	0	0	0	0	0	1 (15)

Translation proteins SH3-like domain	0	0	3 (57^a^)	0	0	0	1 (19)

Ribosomal protein L14	0	0	1 (19^a^)	0	0	0	1 (19)

Nucleic acid-binding proteins	15 (59)	0	2(1+19^a^)	1 (1)	1 (1)	4 (10)	10 (50)

Translation proteins	1 (6)	0	1 (19^a^)	0	0	0	8 (40)

EF-Tu/eEF-1α/eIF2-γ C-terminal domain	1 (6)	0	0	0	0	0	4 (18)

ValRS/IleRS/LeuRS editing domain	2 (7)	0	0	0	0	0	1 (2)

Metallo-dependent hydrolases	9 (50)	2 (9)	2 (4)	2 (32)	1 (2)	2 (2)	2 (4)

HAD-like	1	0	0	1	0	0	14 (35)

Ribosomal proteins L15p and L18e	0	0	2 (38^a^)	0	0	0	2 (38)

PIN domain-like	2 (3)	0	0	0	0	2 (2)	2 (2)

NAD(P)-binding Rossmann-fold domains	13 (89)	0	3 (7)	2 (2)	1 (1)	7 (14)	11 (20)

Initiation factor IF2/eIF5b, domain 3	0	0	0	0	0	0	1 (1)

Ribosomal protein S2	1 (18)	0	0	0	0	0	1 (15)

Class I glutamine amidotransferase-like	3 (4)	0	1 (2)	1 (4)	0	1 (10)	5 (5)

Nucleotidylyl transferase	8 (24)	0	1 (6)	0	0	2 (2)	9 (15)

Adenine nucleotide α hydrolases-like	8 (24)	0	1 (6)	0	0	3 (3)	13 (34)

FAD/NAD(P)-binding domain	1 (1)	0	0	0	0	1 (2)	2 (3)

P-loop containing nucleoside triphosphate hydrolases	18 (45)	0	3 (13)	2 (2)	0	8 (12)	108 (455)

Anticodon-binding domain of Class II aaRS	2 (15)	0	0	0	0	0	1 (4)

Actin-like ATPase domain	2 (3)	0	1 (1)	0	0	1 (1)	7 (38)

Ribonuclease H-like	8 (43)	0	3 (3+19^a^)	0	0	8 (15)	10 (76)

Translational machinery components	1 (18)	0	1 (19^a^)	0	0	0	2 (34)

S-adenosylmethionine-dependent methyltransferases	1 (1)	0	1 (1)	1 (1)	0	0	1 (3)

Class II aaRS and biotin synthetases	2 (15)	0	0	0	0	4 (4)	5 (13)

ATPase domain of HSP90 chaperone/DNA topoisomerase II/histidine kinase	0	0	0	0	0	1 (2)	11 (25)

Creatinase/aminopeptidase	2 (3)	0	0	0	0	2 (8)	0

DNA clamp	1 (1)	0	0	0	0	0	1 (1)

Ribosomal protein S5 domain 2-like	4 (39)	0	2 (2)	0	0	0	12 (49)

Ribosomal protein S8	1 (19)	0	0	0	0	0	1 (15)

Ribosomal protein L6	0	0	1 (19^a^)	0	0	0	1 (19)

DNA ligase/mRNA capping enzyme, catalytic domain	2 (3)	0	0	0	0	1 (1)	1 (1)

Metallo-hydrolase/oxidoreductase	4 (33)	1 (1)	2 (1+2^b^)	0	0	1 (1)	0

FKBP-like	4 (4)	1 (1)	0	0	0	0	0

Ribosomal protein L10e			1 (19^a^)	0	0	0	1 (19)

dsRNA-binding domain-like	1 (18)	0	0	0	0	0	1 (15)

Ribosomal protein L22	0	0	1 (19^a^)		0	0	1 (19)

EF-G/eEF-2 domains III and V	0	0	0	0	0	0	2 (2)

α-L RNA-binding motif	1 (18)	0	0	0	0	0	1 (15)

Threonyl-tRNA synthetase (ThrRS), second 'additional' domain	1 (3)	0	0	0	0	0	0

RBP11-like subunits of RNA polymerase	3 (21)	0	0	0	0	2 (14)	3 (7)

Ribosomal protein L5	0	0	1 (19^a^)	0	0	0	1 (20)

Ribosomal protein L1	0	0	0	0	0	0	1 (1)

β and β' subunits of DNA dependent RNA-polymerase	4 (22)	0	0	0	0	2 (14)	4 (10)

Fold superfamily names are from the SCOP database [174]; metal content is from the Protein Data Bank [140]. The figures show the non-redundant numbers of hits, the figures in parentheses show the total number of hits (see text for details).

a – Proteins of the large ribosomal subunit, whose crystals were soaked in CdCl_2 _during sample preparation ([147,148], 19 different PDB entries).

b – Cd-substituted Zn-dependent metallo-beta-lactamase [175].

##### Occurrence of Zn in the proteins with the oldest functions

The Zn world concept, as well as some other evolutionary scenarios (see [28] and references therein) imply that the first enzymes could have emerged to overtake the catalytic functions from ribozymes. An up-to-date list of the known catalytic activities of ribozymes can be found in ref. [29]. In the absence of proteins or organic cofactors, ribozymes, especially the artificial ones, can catalyze breakdown and formation of diverse covalent bonds, as well as group transfer reactions by employing acid-base catalysis which often proceeds in a metal-assisted way (see the discussion above and refs. [29,35,151-155,176]). The first enzymes can be expected to have had similar catalytic activities. If the first enzymes emerged in the Zn-rich environments, one could expect involvement of Zn^2+ ^ions in the oldest enzymes, particularly those that catalyze the formation and breakdown of covalent bonds and the group transfer reactions. This consideration leads to the prediction no. 4: *The enzymes with evolutionarily "oldest" functions, including catalysis of formation and breakdown of chemical bonds, should depend on zinc*.

Zerkle and co-workers recently analyzed biogeochemical signatures, trying to reconstruct changes in the enzyme metal content in the course of evolution [125]. Their reconstruction showed that 37% of metalloenzymes that could be timed to the "very early life" were Zn-dependent with their relative fraction dropping to 19% in modern organisms. The fraction of Mn-dependent enzymes remained almost constant (10% versus 9%), while the fraction of iron-dependent enzymes increased from 18% to 34%. These data [125] indicate that presumed evolutionarily oldest functions were largely performed by Zn-containing enzymes.

To check the prediction on zinc dependence of the enzymes that catalyze formation and breakdown of chemical bonds, as well as group transfer reactions, we have analyzed the involvement of transition metals as cofactors in different enzyme classes according to the enzyme descriptions in the International Union of Biochemistry and Molecular Biology (IUBMB) Enzyme Nomenclature, as documented, among others, in the ExporEnz database [177,178]. Since we needed specific information on metals as catalysts, and not just as structural elements, we searched the MACiE (Mechanism, Annotation and Classification in Enzymes) database [179,180] and its recently described Metal-MACiE supplement, a database of metal-based reaction mechanisms [181,182]. Andreini and co-workers [181] compared metal-containing enzymes listed in these databases with metal-containing proteins in the PDB and showed that the relative occurrence of catalytic metals in Metal-MACiE matched well that in the PDB. Table 3 shows the presence of metal cofactors in different groups of enzymes listed in Metal-MACiE database (complemented by information from the MACiE database to distinguish between Fe^2+ ^and Fe^3+^).

**Table 3:** Metal dependence of the enzyme catalytic activities.

Enzyme class	Zn	Mn	Fe^2+^	Fe^3+^	Cu	Co	Ni	Mo
Oxidoreductases	1	1	33	6	6	1	1	7

Transferases	1	5						

Hydrolases	17	4	1^a^	1			1	

Lyases	6	2	1	1		1		

Isomerases		2				5		

Ligases								

The numbers of enzymes with particular transition metals as catalytic cofactors are from the MACiE database [179,180], and its recently described Metal-MACiE supplement [181,182], see text for details.

^a ^– Peptide deformylase, where the Fe atom is functionally replaceable by Co, Ni or Zn [185].

As follows from Table 3, Fe, Cu and Mo are catalytically active in oxidoreductases (which catalyze electron transfer reactions), whereas Zn^2+ ^ions are the predominant metal cofactors in hydrolases (which catalyze breaking of chemical bonds with the involvement of a water molecule) and lyases (which catalyze breaking of various chemical bonds by means other than hydrolysis and oxidation). For transferases (which catalyze transfer of functional groups from one molecule to another), the preferred metal cofactor was Mn. Only few hits were obtained for isomerases, with a preference for Co as a cofactor. As seen in Table 3, no transition metal hits were obtained for ligases (which catalyze joining of two molecules by forming a new chemical bond), although, according to the MACiE database, some ligases depend on Mg^2+ ^ions. Since the number of hits in the Metal-MACiE database was small for transferases, isomerases and ligases, the survey of these enzyme classes was expanded by extracting additional data from BRENDA (BRaunschweig ENzyme DAtabase, [183-185]). BRENDA is manually curated and contains a wealth of information on the properties of various enzymes, including presence of metals and their likely functions [183,184]. In the case of transferases, the involvement of Zn^2+ ^ions as cofactors seemed to be less specific than of Mn^2+^; in the vast majority of cases, both Zn^2+ ^and Mn^2+ ^ions could be functionally replaced by other divalent cations such as Mg^2+^, Ni^2+ ^or Co^2+^. Involvement of Fe^2+ ^ions in the catalysis by transferases appeared to be limited to their ability to replace Mg^2+^, Mn^2+^, Zn^2+^, Ni^2+ ^or Co^2+ ^ions; Fe^2+ ^ions were routinely reported to be the least efficient catalysts in the series. The list of metal-dependent isomerases shows non-specific utilization of several divalent cations such as Mg^2+^, Mn^2+^, Co^2+^, Zn^2+^, or Ni^2+^. In many cases, the highest enzyme activity, as compared to other cations, was reported with Mn^2+ ^or Co^2+ ^ions, similarly to the data in Table 3. Specific involvement of iron has been shown only for lysine 2,3-aminomutase, where a FeS redox cluster is involved in electron exchange with the catalytic site [186]. Ligases generally use divalent cations, such as Mg^2+^, Co^2+^, Zn^2+^, or Mn^2+^. No evidence of specific catalytic activity of Fe in ligases could be obtained from the BRENDA database.

It is noteworthy that only two transition metals, namely Zn and Mn, are found in the representatives of all six enzyme classes [181]. Zn is by far the most abundant catalytic transition metal in hydrolases and lyases, whereas Mn seems to be involved in transferases and, together with Co, in isomerases. In transferases, isomerases and ligases, the pattern of the transition metal use, with a non-specific need for a divalent cation as catalyst, resembles the catalytic preferences of ribozymes (see above). The Fe^2+ ^ions, with few exceptions, are not used in catalysis outside the oxidoreductases.

Altogether, data on metal content of proteins are consistent with the notion that the early evolution of enzymes could have proceeded in habitats that were enriched in Mg, Zn, and Mn, but depleted of Fe.

#### The elevated Zn^2+ ^content inside modern cells

If life emerged in the environments that had relatively high levels of Zn^2+ ^ions [97], the primordial life forms, which lacked the tools to alter their ionic content [187], should have had high Zn^2+ ^levels as well. In accordance with chemical continuity principle, the ionic content of the primordial environments should be conserved inside modern cells. This leads to the prediction no. 5: *The total amount of Zn*^2+ ^*in the live cell should be elevated as compared to the levels of other essential transition metals*.

Early studies on the total Zn content in several bacteria produced values of ~0.03% of the dry weight, much higher than for any other transition metal except for Fe [188]. The content of Zn, as compared to Fe, was somewhat smaller in *Escherichia coli*, but 2–3 times higher in *Micrococcus roseus *and *Bacillus cereus *[188]. The data for *E. coli *were recently confirmed by inductively coupled plasma mass spectrometry analysis of whole-cell lysates, yielding values of ~200 μM for Zn and 200–300 μM for Fe, depending on the growth conditions [189]. The total Zn content in the human body tissues is, on average, somewhat higher than that of Fe, 3–5 mg versus 2.5–5 mg/100 g of tissue (the data were obtained from tissue samples that had been washed from blood; the amount of other transition metals was much lower [190]). It is noteworthy that although cells contain comparable *total *amounts (100–300 μM) of Fe [191,192] and Zn (see [193] and references therein), the concentrations of *free *(labile) ions differ dramatically, with the free Fe^2+^/Fe^3+ ^concentration of ~10 μM [191,192] and free Zn^2+ ^present only in picomolar amounts [130,193]. These data indicate that intracellular Zn levels are tightly controlled; they also suggest that modern cells are more limited in Zn than in Fe.

Modern sea water contains somewhat more iron, (about 5 nM of mostly Fe^3+^) than Zn^2+ ^(< 2 nM), see [104] and Table 1 in the accompanying article [97]. Hence, compared to the composition of sea water, Zn appears to be the transition metal that is concentrated to the highest extent in the cell. As noted by Williams and Fraústo da Silva, the primeval anoxic ocean must have contained Fe^2+ ^ions, which are more soluble than Fe^3+ ^ions. The available estimates of Fe^2+ ^content in primordial waters are in the range of 10^-6^-10^-5 ^M, compared to the estimate of 10^-15^-10^-12 ^M for Zn^2+ ^ions [124-126]. Hence, the intracellular Zn concentration of 100–300 μM reflects a very efficient scavenging of Zn^2+ ^ions and is consistent with the idea that the emergence of first life forms indeed occurred in very special, Zn^2+^-rich environments.

#### The metallome of the Last Universal Common Ancestor

While the idea that life originated – and the first RNA and protein molecules evolved – in Zn^2+^-rich settings appears to be compatible with the available data, it does not explicitly state whether these Zn^2+^-rich settings played any direct role in the formation of the first cells. Although it is hard to make any specific and verifiable predictions for these matters, in this section we try tracing the possible roles of Zn^2+ ^ions at the times of the Last Universal Common Ancestor of all living cellular organisms (LUCA).

All cellular life forms belong to one of the three main branches of the Tree of Life, Bacteria, Archaea, or Eukarya [194]. The conservation of a set of essential genes between the three domains of life has been considered as evidence in favor of the existence of the LUCA, see [195-197] for reviews. Some researchers view LUCA as a consortium of replicating entities which shared a common gene pool [195]. Alternatively, representatives of the LUCA were suggested to be full-fledged organisms comparable to modern prokaryotes [198,199]. There are also numerous possible variants between these two extreme visions of the LUCA. The infrequency of inter-domain transfer of genes responsible for information processing [200-203] might indicate that these genes, at least at the LUCA stage, already formed constant genetic cores of the first organisms. At the same time, the easily spreading metabolic genes could form a common pool of transferable operational genes [200], such that the organisms, depending on their metabolic requirements, could acquire the necessary tools from a common gene pool. The universal conservation of membrane-embedded subunits of the general protein secretory pathway [204] and the F- and A/V-type ATP synthases [205] has been considered as an indication that the LUCA was already a membrane-encased life form [206]. Its membranes, however, had to be permeable to enable the exchange of genes, proteins and metabolites [73]. The recent modeling by Szathmáry and co-workers showed that "collective" metabolism, with different replicators contributing different metabolites to the common pool, could be a pre-condition for the viability of the whole consortium and its resistance to parasites [207].

Koonin and Martin have argued that the LUCA consortia might have dwelled in networks of iron-sulfur inorganic compartments ("bubbles") of hydrothermal chimneys [138]. As discussed in the accompanying article [97], this model fits nicely into the Zn world concept, provided that the deep-see chimneys built of FeS are replaced by "spongy" ZnS precipitates encircling the sub-aerial hot springs. Precipitation of ZnS at the sites of geothermal activity should have led to continuous formation of new, empty compartments, so that the more competitive consortia could overcome others by "moving in" first. As argued elsewhere [73,138,208], such a scheme implies an extensive (gene) exchange between the members of one consortium, but not between dwellers of different, physically discrete inorganic compartments. It therefore resolves a major conundrum between the notion of extensive gene mixing that is considered a major feature of early evolution [195] and the requirement of separately evolving units for the Darwinian selection.

To what extent the conclusions on primordial bioinorganic chemistry that we have drawn from the data on metal content in modern cells could be related to the LUCA? The intracellular Zn^2+ ^concentration of 10^-3^–10^-4 ^M is a feature that is shared by representatives of all there domains [188-190,209]. The simplest way to explain this remarkable trait is by assuming that the LUCA still lived in Zn-rich habitats. An alternative explanation would assume that the Zn content of LUCA was low and then independently increased in all three major lineages, responding e.g. to the elevation of environmental Zn^2+ ^level from 10^-12^-10^-15 ^M in the anoxic ocean up to 10^-9 ^M after its oxygenation [124-126]. The latter possibility, however, appears unlikely. The high total Zn content in cells is contributed not by free Zn^2+ ^ions, which are scarce [130,193], but by large number of Zn-binding proteins mostly involved in processing of RNA and DNA. These proteins are widespread in all three domains of life and their Zn-binding motifs (in particular, so called "zinc fingers") are homologous [210]. Therefore it appears unlikely that these Zn-binding motifs could independently develop in different lineages. Since the ligand chemistry of these binding sites is specifically tuned to prefer Zn^2+ ^over other transition metal ions [211,212], it is equally unlikely that they served first to bind some other metal, e.g. iron, and only later adapted to binding zinc. It also appears implausible that selective Zn-enrichment of LUCA's interior could be accomplished by powerful ion pumps capable of maintaining the huge Zn gradient between the LUCA's interior and the surrounding Zn-depleted, anoxic waters. As argued elsewhere, the LUCA should have had primitive membranes [73,138,187,206] that could not hold the required Zn concentration gradient of > 10^8^; even the modern membranes can hardly do that. Thus, the most parsimonious explanation of the high cellular content of Zn in representatives of all three domains of life is by suggesting that the LUCA thrived in Zn-rich habitats that apparently were in the ionic equilibrium with the LUCA's interior.

It is tempting therefore to make prediction no. 6: *The proteins that could be attributed to LUCA should be enriched in Zn*. The problem of the LUCA-specific protein set has been addressed by several authors, see [197] for a review. After completion of the first microbial genomes, a "minimal" set of genes shared by these genomes was deduced; it has been speculated that these genes made the genome of the LUCA [213,214]. With increasing number of sequenced genomes, the set of genes shared by all genomes kept shrinking; it has become clear that with just ~50 such genes it would not be possible to build a full-fledged organism [196,215]. Accordingly, it is now believed that the metabolism of the LUCA was carried out by operational genes that are not necessarily conserved in all genomes [216]. However, a small set of genes that are shared by all known genomes is still believed to form the core of the LUCA's genome [196,197,215]. The products of these ubiquitous genes and their metal affinities are listed in Table 4 [217-238] and show a notable preference for Zn and Mg as metal cofactors. Iron was found only in some structures of a single protein family (YgjD/Gcp/QRI7) of obscure function that was originally reported to have O-sialoglycoprotein endopeptidase activity, later identified as an apurinic endonuclease, and recently shown to be essential for genome maintenance in Archaea and Eukarya [234,239,240]. These data indicate that proteins likely to be present in the LUCA – and, hence, the LUCA itself – existed in a Zn-rich environment. As discussed above, since the equilibrium Zn concentration in the primordial oceans must have been extremely low [124-126], a Zn rich environment could persist only due to some steady geochemical reaction leading to continuous release of Zn^2+ ^ions, such as abiogenic photosynthesis.

**Table 4:** Association of essential divalent metals and the products of ubiquitous genes.

Protein function	EC number (if available)	Functional dependence on metals	Metals in at least some structures
**Products of ubiquitous genes, according to **[196]			

**Translation and ribosomal biogenesis**			

Ribosomal proteins (33 in total)		Mg	Mg, Zn (see main text)

Seryl-tRNA synthetase	6.1.1.11	Mg, Zn	Mn [218], Zn [219]

Methionyl tRNA synthetase	6.1.1.10	Mg, Zn	Zn [220]

Histidyl tRNA synthetase	6.1.1.21	Mg	No metals seen

Tryptophanyl- tRNA synthetase	6.1.1.2	Mg, Zn	Mg

Tyrosyl- tRNA synthetase	6.1.1.1	Mg	No metals seen

Phenylalanyl- tRNA synthetase	6.1.1.20	Mg, Zn	Mg

Aspartyl- tRNA synthetase	6.1.1.12	Mg	Mg, Mn [221]

Valyl-tRNA synthetase	6.1.1.9	Mg	Zn [222]

Isoleucyl-tRNA synthetase	6.1.1.5	Mg, Zn	Zn [223,224]

Leucyl-tRNA synthetase	6.1.1.4	Mg	Zn [225]

Threonyl-tRNA synthetase	6.1.1.2	Mg, Zn	Zn [226]

Arginyl-tRNA synthetase	6.1.1.19	Mg	No metals seen

Prolyl-tRNA synthetase	6.1.1.15	Mg, Zn	Mg, Zn [227], Mn [228]

Alanyl-tRNA synthetase	6.1.1.7	Mg, Zn	Mg, Zn [229]

Translation elongation factor G	3.6.5.3	Mg	Mg

Translation elongation factor P/translation initiatiation factor eIF5-a			Zn (PDB entry 2E9H)

Translation initiation factor 2			Zn [230]

Translation initiation factor IF-1			No divalent metals

Pseudouridylate synthase	5.4.99.12	Mg, Zn [217]	No metals seen

Methionine aminopeptidase	3.4.11.18	Mn, Zn, or Co	Mn or Zn or Co

**Transcription**			

Transcription antiterminator NusG	-	-	No metals seen

DNA-directed RNA polymerase, subunits α, β, β'	2.7.7.6	Mg	Mg, Mn, Zn [231]

**Replication**			

DNA polymerase III, subunit β	2.7.7.7	Mg	Mg [232]

Clamp loader ATPase (DNA polymerase III, subunit γ and τ)	2.7.7.7.	Mg	Mg, Zn [233]

Topoisomerase IA	5.99.1.2	Mg	No metals seen

**Repair and Recombination**			

5'-3' exonuclease (including N-terminal domain of PoII)	3.1.11.^a^	Mg	Mg

RecA/RadA recombinase	-	-	Mg

**Chaperone function**			

Chaperonin GroEL	3.6.4.9	Mg	Mg

O-sialoglycoprotease/apurinic endonuclease	3.4.24.57	Zn [239]	Mg, Fe [234]

**Nucleotide and amino acid metabolism metabolism**			

Thymidylate kinase	2.7.4.9	Mg	Mg

Thioredoxin reductase	1.8.1.9	-	No metals seen

Thioredoxin		-	Zn [235]

CDP-diglyceride-synthase	2.7.7.41	Mg	No entries

**Energy conversion**			

Phosphomannomutase	5.4.2.8	Mg	Mg, Zn [236]

Catalytic subunit of the membrane ATP synthase	3.6.1.34	Mg	Mg

Proteolipid subunits of the membrane ATP synthase	3.6.1.34	-	No metals seen

Triosephosphate isomerase	5.3.1.1	-	No metals seen

**Coenzymes**			

Glycine hydroxymethyltransferase	2.1.2.1	Mg	No metals seen

**Secretion**			

Preprotein translocase subunit SecY	-	-	Zn [237]

Signal recognition particle GTPase FtsY	-	-	Mg

**Miscellanous**			

Predicted GTPase	-	-	No metal ligands in the structures

**Additional ubiquitous gene products from ref. **[215]			

DNA primase (dnaG)	2.7.7.-	-	Zn [238]

S-adenosylmethionine-6-N', N'-adenosyl (rRNA) dimethyltransferase (KsgA)	2.1.1.48	Mg	No metals seen

Transcription pausing, L factor (NusA)	-	-	No metals seen

The lists of ubiquitous genes were extracted from refs. [196,215]. The data on metal dependence were taken from the BRENDA database [185], the presence of metals was as listed in the Protein Data Bank [140] entries. According to the BRENDA database [185], the enzymatic activity of most Mg-dependent enzymes could be routinely restored by Mn. As concentration of Mg^2+ ^ions in the cell is ca. 10^-2 ^M, whereas that of Mn^2+ ^ions is ca. 10^-6 ^M, the data on the functional importance of Mn were not included in the table.

*Summarizing the preceding part of the Results*, Zn-rich habitats appear to have shaped the primeval biochemistry by favoring the emergence of Zn-stabilized protein and RNA folds, as well as Zn-dependent enzymatic reactions. Seemingly, the development of the first life forms proceeded in the Zn-rich settings up to the stage of the LUCA.

### Testing the Explanatory Power of the Zn world concept

The Zn world concept offers an entirely new look at many aspects of the primeval evolution and biochemistry. Tracking all the facts that could be explained by the Zn world concept better than by other hypotheses on the origin of life is beyond the scope of this paper. In particular, we anticipate that this concept will provide a framework for many observations related to the biochemistry of zinc. Here we consider only those explanations that clearly separate the suggested concept from other hypotheses of abiogenesis. Besides, we try focusing on the phenomena that until now did not have acceptable explanations.

#### Energetics of abiogenesis

The Zn world concept explains how both the reduction of CO_2 _and the primeval biosyntheses could be driven by solar energy (see the accompanying article [97] for details). Other hypotheses on the origin of life either do not consider the energetics of abiogenesis explicitly (heterotrophic origin of life/RNA World) or, as the above discussed concepts of Wächtershäuser [56-61] and of Russell and co-workers [62-71], suggest mechanisms that do not seem to be plausible from the physical or (bio)chemical viewpoints (see [72] and the Background section above).

#### Photostability of polynucleotides

As discussed in the accompanying article [97], (poly)nucleotides, especially those building Watson-Crick pairs, are uniquely photostable (see also [33,120,121]). The Zn world concept explains this unique photostability by the role of the UV light not only as an energy source, but also as a selective factor during the first evolutionary steps. The unique photostability of (poly)nucleotides finds no explanation in any other hypothesis on the origin of life.

#### The zinc paradox

As discussed above, Zn^2+ ^ions are used as cofactors by several groups of enzymes that are mostly involved in the cleavage or formation of chemical bonds (see Section 2.1.2.3). Thereby Zn^2+ ^ions serve as Lewis acids upon catalytic transitions [128]. Generally, the capacity of a transition metal to serve as a Lewis acid is determined by its position in the Irving-Williams series and should increase as: Mn < Fe < Co < Ni < Cu > Zn [241]. Hence Zn, as a Lewis acid, is expected to be better than Mn or Fe, but worse than Cu. However, as specifically noted by Williams and Fraústo da Silva [128], the difference between the transition metals in this respect is not that great, and deviations from the Irving-Williams series are possible, e.g. owing to the influence of the enzyme ligands. In many experiments, Zn atoms could be replaced by other transition metal atoms with only minor loss in the enzyme activity (in some cases, even with an increase in activity) [128,242]. Therefore, the almost exclusive involvement of Zn as cofactor in all these enzymes has been considered enigmatic, especially taking into account the low levels of Zn in the seawater [124,128]. Moreover, while prevalence of Zn in certain types of enzymes could be attributed to the catalytic properties of Zn^2+ ^ions, their ubiquitous involvement as structural elements [128,210,243] had no explanation at all. This paradoxical prevalence of Zn ions can now be explained by the shaping – and folding – of first proteins in Zn-rich habitats.

*Summarizing this section*, we can conclude that the Zn world concept offers a single parsimonious explanation for a set of diverse observations that have not been rationalized so far.

## Discussion

In this work, we made six non-trivial biological predictions stemming from the idea of the origin of life in Zn-rich settings. Specifically, we predicted that Zn^2+ ^ions would be preferentially associated with ancient RNA and protein molecules, including ribozymes and those enzymes that catalyze evolutionarily old reactions. These predictions were tested using publicly available data, obtained in studies that had no apparent bias towards Zn. The results of these tests revealed that modern cells contain surprisingly high levels of Zn, which is mostly bound to its constituent molecules, DNA, RNA and proteins. The most parsimonious explanation of these observations seems to be that, indeed, the first life forms evolved in a Zn-rich environment.

In addition, following the Popper's principles, we have tested the Zn world concept by considering the ability of this concept to provide explanations for obscure facts that other theories either ignore or cannot explain. The fact that the Zn world concept has successfully passed all these tests makes it a serious contender for the title of a syncretic concept of the origin of life.

### Zinc world: No country for old iron?

Some of the results obtained in the course of this work were rather unexpected, for example, the almost complete absence of the Fe atoms in the evolutionarily oldest protein folds (Table 2) and in putative proteins of the LUCA (Table 4). The apparent absence of correlation between the supposedly primitive traits of life forms and the involvement of iron, which could be seen in a variety of tests (Tables 1, 2, 3, 4), strongly argues against the view that life has emerged in iron-rich environments [56-58,61-71,77,136]. This iron-centric view is based, among others, on the fact that the iron-sulfur clusters could serve both as protein cofactors (e.g. in ferredoxins) and crystal units of natural minerals (see [67,244] and references therein). The argumentation, however, could be equally well applied to Zn. Zinc atoms and ZnS clusters are prevalent both in hydrothermal settings [104,117] and, as cofactors, in proteins [128,245-247]. Furthermore, proteins that coordinate either Zn atoms or ZnS clusters seem to be more widespread than iron-sulfur proteins [130,245-251]. Ironically, the first zinc-sulfur protein, metallothionein, had been described by Margoshes and Vallee [252] even before the discovery of the first iron-sulfur protein, ferredoxin [253]. Sequence similarities between proteins that bind FeS and ZnS clusters were noted e.g. by Williams and Fraústo da Silva [128]. Some metal-binding protein scaffolds can bind either Fe or Zn, depending on their relative concentrations (see [254,255] and references therein). Remarkably, the iron-sulfur cluster assembly protein IscU is capable of binding Zn^2+ ^ion in its monomeric form [256], whereas three such monomers have to interact to bind a FeS cluster [257]. The Zn-binding mode could well be the evolutionarily older one in this protein. While FeS clusters are involved, to a large extent, in electron transfer reactions (see Table 3), zinc-sulfur proteins are mostly associated with RNA and DNA, e.g. as zinc fingers [243,245,246,250]. In the view of the assumed evolutionary primacy of RNA, one could imagine that, in a Zn-rich environment, zinc-sulfur proteins could have emerged first. In fact, it is extremely unlikely that FeS clusters could have ever been directly involved with RNA since they are efficient cleavage agents for both RNA and DNA (see [258] and references therein), not to mention hazardous hydroxyl radicals that could be produced in the presence of redox-active Fe^2+^/Fe^3+ ^ions [168-170]. The Fe atoms and FeS clusters could replace Zn atoms and ZnS clusters – in some cases – only after the emergence of enzymes and membranes which could protect RNA and DNA from the damaging action of iron and its compounds. The redox-active Fe and Cu atoms could be recruited as redox cofactors (in support to the nucleotide-based cofactors such as NAD(P)H, FMN, FAD, see the accompanying article [97] and references therein) by enzymes of those energy-converting systems that eventually replaced the ZnS-mediated photosynthesis. This time pattern is in agreement with the results of the above discussed analysis of the changes in biogeochemical signatures through time [125], where the relative fraction of Zn-dependent enzymes decreased in the course of evolution, whereas the fractions of the Fe- and Cu-dependent enzymes have increased. The importance of redox enzymes must have further increased with the oxygenation of Earth habitats, such that the total content of Fe in modern organisms is compatible with that of Zn.

### Zinc world: Metals and first biotopes

Testing the predictions on metal binding by RNA and protein molecules also revealed a notable presence of Mn atoms in RNA structures and the oldest protein folds. This presence of Mn might be not accidental. Manganese is unique in at least two respects:

a) Mn^2+ ^ions are typical constituents of hydrothermal fluids [259,260]. In experiments that modeled the high-pressure conditions at hydrothermal vents, MnS precipitated at the same rate as ZnS, i.e. much slower than sulfides of Fe and Cu [259]. Hence, one can expect that the sulfides of Zn and Mn could precipitate at approximately the same distance from the orifices of the primeval sub-aerial hot springs and could form mixed ZnS/MnS haloes around them, as found in the ancient volcanogenic metal sulfide (VMS) deposits where the haloes of neighboring vents intersect and join into networks [105,261].

b) MnS is the only other transition metal sulfide – besides ZnS – that can photoreduce CO_2 _albeit, seemingly, with a lower quantum yield (see Fig. 1 and refs. [95,262]). The band gap of MnS is smaller than that of ZnS, about 3–3.5 eV versus 3.2–3.9 eV (see Fig. 1 and [262-264]). Because of the smaller band gap, MnS can photoreduce CO_2 _by using visible light.

Hence, photoactive formations that contained MnS in addition to ZnS could use for photosynthesis not only the UV quanta, but also the visible light (up to ca. 450 nm), which could increase the productivity of the first photosynthetic communities. In addition, because of its lower scattering, visible light could penetrate deeper into the porous interior of the photosynthetic edifices. We would like to note that the possible supportive role of Mn in the Zn world did not follow from the premises of the original hypothesis [97], but transpired during its testing.

The Zinc world concept provides a plausible answer to the question why some transition metals are essential for living organisms while others are not. The less frequent – as compared to Zn and Mn – usage of other transition metals as cofactors can be explained by their scarcity in the settings that hosted the first life forms. Generally, hydrothermal fluids contain not only Zn^2+^, Mn^2+ ^and Fe^2+ ^ions, which we have discussed so far, but also notable amounts of Pb, Cu, Ni, Co and some other metals, with exact composition varying depending on location [104-106,260]. These metals are also found, in variable amounts, in the ancient VMS deposits (see [261,265] for reviews and the accompanying article [97] for further details and references). However, the only metal sulfides that can photoreduce CO_2 _are ZnS and MnS (see [95,99,122,262,264]). Other transition metals, if present as substantial impurities in the ZnS/MnS settings, would function as energy traps for the photo-excited electrons (see Fig. 1 and ref. [264]) and decrease the quantum yield of the abiogenic photosynthesis. Hence, the exact metal content of sulfide precipitates, most likely, could vary at different spots of primeval hydrothermal activity depending on (i) the chemical composition of the underlying crust, (ii) the temperature of hydrothermal fluids and (iii) their pH value (as it varies nowadays, see [104-106,260]). However, only those precipitates made of ZnS and MnS could photosynthesize, support the first organisms, and, hence, be inhabited. Accordingly, if we consider a particular transition metal ion, the probability of its recruitment for some primeval biochemical task could be proportional to its concentration at a particular habitat multiplied by the number of potential "recruiters", i.e. the life forms present. As a result, some photosynthetically inert transition metals became involved only occasionally (e.g. Co, see Table 3 and ref. [266]) or upon later evolutionary steps (as Fe, see discussion above), whereas others failed to attain any essential biological function (e.g. Pb).

The suggested concept might also explain why aluminum, although widespread in the Earth crust and soil, has not been recruited for biochemical tasks. The sulfides of aluminum, as well as of titanium, are unstable in water. Therefore aluminum does not precipitate at the spot of hydrothermal activity but becomes dissolved in water and apparently comes down later, far away from the hydrothermal orifices [267]. The absence of aluminum among essential metals, when combined with the importance of sulfur for biochemistry, appears to discount those models of abiogenesis that envision the origin of life in clays (see [268] and references therein) since clays are aluminum silicates that, unlike hydrothermal sulfide precipitates, do not contain sulfur.

**Figure 2:**
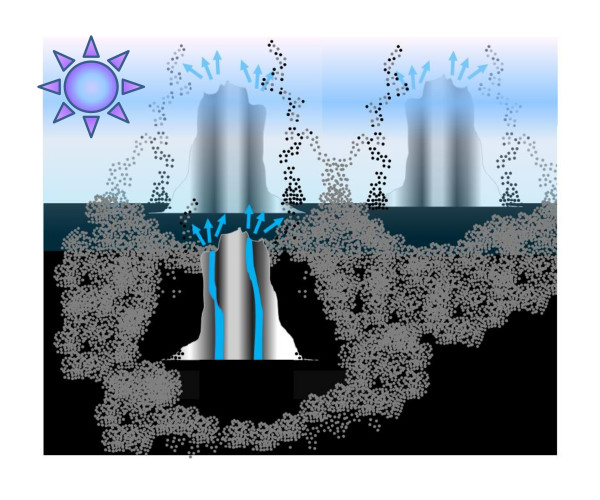
**A schematic representation of interweaved haloes made of porous ZnS/MnS (shown as aggregates of grey spheres) around the sub-aerial, hydrothermal hot springs**. These networks are proposed to have served as the Earth's first biotopes (see the text and the accompanying article [97]). The picture uses data from refs. [66,105,115,117,260,261].

Based on available geochemical data, in particular on the architecture of the ancient VMS deposits [105,261,265], one can envision networks of photosynthesizing and habitable bands of precipitated ZnS and MnS around primeval hot springs. These networks of joined rings at the spots of geothermal activity, a kind of primeval "Yellowstone Park" realm, could represent the first Earth biotopes (see Fig. 2).

### Decline and fall of the Zinc world

We can only speculate on the sequence of events that followed the drop in the atmospheric pressure below 10 bar and the gradual decay in the delivery of hot, Zn-rich hydrothermal fluids to the illuminated settings. An obvious consequence of these developments was gradual vanishing of illuminated ZnS surfaces and cessation of abiogenic photosynthesis.

**Figure 3:**
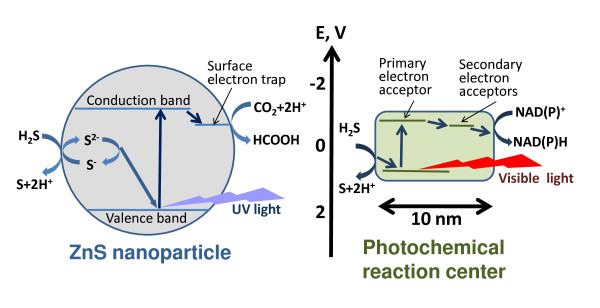
**A comparison of energy diagrams for a photosynthesizing ZnS nanoparticle (left panel, the picture is taken from the accompanying article **[97]** and is based on references **[98,103,122]) **and a bacterial photochemical reaction center (right panel, a primitive, sulfide-oxidizing reaction center complex of green sulfur bacteria **[276,277]** is shown schematically as an example)**.

As suggested in the accompanying article [97], ZnS-dependent communities should have been functionally stratified, just as the modern phototrophic communities are (see [45,269] and references therein). If so, the inhabitants of different ZnS strata would encounter vastly different levels of UV irradiation and evolve under different selective pressures. In particular, those inhabiting upper, light-exposed layers would need some protection from the damaging UV light. Such protection could be provided by UV-absorbing porphyrins and/or chlorins (precursors of chlorophylls). Being attached to proteins, these rings could convert the UV quanta, after absorbing them directly or getting them from nearby aromatic amino acids, into harmless red quanta [270]. In response to the demise of the ZnS-mediated photosynthesis, the life forms in the upper layers could use their chlorin-containing proteins to catalyze light-driven separation of electric charges [270] and thus become capable of reducing such compounds as e.g. NAD(P)H, which could then convey the electrons to metabolic chains. Sequence and structural analyses showed that modern photochemical (photosynthetic) reaction centers could have emerged from dimerization of the ancestral simpler chlorin-carrying membrane proteins [270-275], which, in turn, could function as UV-protectors of primordial cells [270]. Halmann and colleagues [81] noted the similarity between physical mechanisms of the chlorophyll-based and semiconductor-based photosyntheses, which both include light-induced charge separation followed by the stabilization of the reduced states, as shown in Fig. 3. This figure also shows that if we focus on the photosynthesis by ZnS nanoparticles, even the sizes of the abiogenic and biogenic photochemical devices match each other. In addition, the same reaction of sulfide/sulphur oxidation is used to re-fill the photo-generated electron vacancies (holes) in the most primitive, homodimeric photochemical reaction centers of green sulfur bacteria [276,277]. The disadvantage of the modern protein-based photoreaction centers, as compared to ZnS crystals, is that they cannot reduce CO_2 _directly. Therefore a full-fledged protein-based photosynthesis must include some version of the Calvin cycle to incorporate CO_2 _into organic molecules at the expense of photoreduced NAD(P)H. As argued by Gánti [278], the Calvin cycle could develop directly from the Butlerov's reaction [279-281], since the sugar intermediates of the Calvin cycle essentially overlap with the components of this autocatalytic pathway (see also the accompanying article [97] and references therein). The biogenic photosynthesis could initially complement the gradually diminishing ZnS-mediated photosynthesis; its contribution, however, should have increased with time, as the formation of the ZnS surfaces in the illuminated, sub-aerial settings came to the end. In this framework, the emergence of biogenic photosynthesis might represent a clear-cut case of functional takeover – with the primeval photochemical reaction centers and primordial Calvin cycle accomplishing together the function that the ZnS/MnS covered fields could perform alone, namely the utilization of solar energy for fixation of CO_2 _(see Fig. 3).

Hence, although after the drop in the atmospheric CO_2 _pressure the photosynthesizing ZnS edifices could no longer build up in the illuminated settings, the life forms could persist in these habitats by relying on the protein-based photosynthesis. In the absence of the ZnS settings, the organisms, however, had to undergo major changes upon adapting to the new environments. This selective pressure should have favored formation of encased, bacteria-like entities that could maintain – in their interior – the chemical content similar to that in the Zn world, i.e. the high Zn level needed for the RNA and DNA processing machinery (see the previous sections). Since the concentration of Zn ions in the sea water is low, these organisms had to develop active membrane ion pumps to maintain high Zn levels in their interior, see [282] for reviews.

The Zn world, however, did not vanish completely; fresh, porous ZnS edifices continued to build up at the sea floor, owing to the high temperature of the deep-sea hydrothermal fluids. These ZnS habitats could still accommodate life forms, which, however, could no longer rely on abiogenic photosynthesis. One possible metabolic strategy for such organisms would be chemoautotrophy, i.e. obtaining reducing equivalents and energy from oxidation of sulfide or hydrogen, the approach which they could already practice while thriving in the dark, bottom layers of the photosynthesizing ZnS settings and which the prokaryotic inhabitants of hydrothermal vents still use these days. This strategy would impose strict limits on the size of living organisms, as they would have to maintain a high surface-to-volume ratio [283]. These organisms could gradually spread away from the ZnS settings and populate iron-rich settings, provided that they developed the cell envelopes and other tools to keep the intrinsic Zn level high.

The most conservative survival strategy would be to remain confined to the ZnS edifices and to retain the ancient heterotrophic way of life, i.e. to consume organic compounds – e.g. by using Zn-dependent hydrolases [128,284,285] – that could come with hydrothermal fluids and/or result from the activity of the chemotrophic organisms. From the evolutionary point of view, such heterotrophs remained adherent to the primeval way of life and, hence, could retain some ancient features (e.g. high dependence of their metabolism on Zn).

The accompanying paper [97] starts with Darwin's famous notion that emergence of living substance anew is extremely unlikely because "...at the present day such matter would be instantly devoured or absorbed, which would not have been the case before living creatures were formed [286]". Here we argue that the living matter may have emerged on Earth owing to a unique interplay between the solar UV-light and the geochemical conditions that brought into existence the sub-aerial Zn world. Thus, we dare to suggest that once the photosynthesizing Zn world could not persist anymore – perhaps, partly as a consequence of CO_2 _consumption by the first life forms – there was no force left to power subsequent origins of life on Earth.

### Separation of the Main Domains of Life

The mechanism of separation of LUCA's descendants into three main lineages remains controversial (see [138,287,288] and references therein). Zillig and co-workers have suggested that the split of Bacteria and Archaea resulted from a geographic separation of two populations [289]. Gogarten-Boekels and colleagues proposed a catastrophe, such as a major meteorite impact, with ancestors of Bacteria and Archaea, respectively, as survivors of this catastrophe [290]. Woese suggested a 'genetic annealing' of the common gene pool as a mechanism leading to the three domains of life [195]. Martin and co-workers have suggested that Bacteria and Archaea are descendants of two distinct populations that thrived within an iron-sulfur deep-sea hydrothermal vent [64,138].

Whatever the separation mechanism(s), the modern representatives of the three domains of life are quite different. These differences seem to indicate that they have evolved, after the splitting of the main lineages, under different environmental conditions. Thus, any scenario of the domain separation has to include a tentative explanation of the key differences between the three domains of life. For example, it has to be explained why the (bacterio)chlorophyll-based photosynthesis is found in Bacteria but not in Archaea. Answering this particular question, Nisbet and Fowler hypothesized that (bacterio)chlorophyll-based photosynthesis has developed, among some inhabitants of the deep-sea hydrothermal vents, from heat sensors that could react to infrared radiation. These organisms, after their eventual migration into the sub-aerial habitats, could switch to the photo-autotrophic growth and eventually give rise to future Bacteria [291]. Alternatively, Russell and co-workers hypothesized that after the first life emerged at a deep-sea hydrothermal vent, a geological obduction could bring a portion of the deep-sea biosphere into the photic zone, with (bacterio)chlorophyll-based photosynthesis subsequently emerging in this population [65,68].

The Zinc world scenario, in principle, can explain both the emergence of the main domains of life and the specific traits of the organisms belonging to them. Indeed, as argued above, the LUCA consortia could have inhabited photosynthesizing, porous ZnS settings. In the previous section, we have discussed the possibility that the inhabitants of different layers of the ZnS-confined communities could respond differently to the gradual decay in the ZnS deposition in the illuminated settings. The inhabitants of the upper layers would be switching to the (bacterio)chlorophyll-based photosynthesis, whereas the dwellers of the lower, darker layers would either turn to the chemoautotrophy or, alternatively, become highly specialized heterotrophs. It is noteworthy that with gradual migration of the high-temperature hydrothermal systems – and their inhabitants – into the sea depths, the sub-aerial phototrophic communities would eventually separate from the consortia staying with the hydrothermal vents. This separation would then persist at least until the emergence of the swimming mechanisms that enabled movement in the water column. During this time, discrete lineages would evolve independently and attain their specific traits.

The outlined hypothetical scenario implies that the demise of ZnS mediated photosynthesis triggered a major separation of the first life forms into (i) the sub-aerial communities dependent on (bacterio)chlorophyll-type photosynthesis as source of reducing equivalents (the future Bacteria) and (ii) the communities confined to the ZnS settings at the sea floor. The dwellers of the sea floor habitats could diversify further. Some of their lineages would evolve by developing new types of metabolism, e.g. chemoautotrophy. Acquisition of cell envelopes would enable their spread into Zn-poor media and give rise to diverse archaeal branches. In contrast, the most conservative lineage would remain adherent both to the ancient ZnS milieu and to the primeval, heterotrophic way of life. Only after the dwellers of sub-aerial habitats developed swimming machinery they would have been able to detach from the shoreline and populate the ocean photic zones. A certain mixing between lineages would then become possible, enabling a "lateral" gene exchange between them [292]. The flow of organic matter from the surface water layers to the sea floor, owing to the sedimentation processes, had to be more extensive than in the opposite direction. Due to sedimentation, the larger, heterotrophic inhabitants of the ZnS settings, not being constrained by size limitations, could eventually acquire the representatives of phototrophic sub-aerial communities as endosymbionts. In particular, a symbiosis with respiring α-proteobacteria, the future mitochondria [293], could, perhaps, rescue some of these heterotrophs (hereafter pro-eukaryotes) from oxidation and extinction after the ocean waters became oxygenated [294,295], paving the way to the modern Eukarya (see [287,288,296] and references therein).

The suggested scenario of the domain separation is based on two premises, namely (a) that the demise of the ZnS-mediated photosynthesis would have forced living organisms to search for new sources of energy and (b) that inhabitants of the stratified ZnS habitats could pursue different strategies upon this search. The outlined scenario reproduces the actual differences between the representatives of the major domains and is in agreement with the following observations:

(i) (Bacterio)chlorophyll-based photosynthesis is present in Bacteria but not in Archaea (see [297] and references therein);

(ii) Inhabitants of the primordial microbial community at the Buck Reef Chert (a 250- to 400-m thick rock along the South African coast that was produced by phototrophic microbes *ca*. 3.4 Ga ago), have been defined as partially filamentous phototrophs, which apparently used the Calvin cycle to fix CO_2 _[298,299]. The reported absence of traces of life in the layers that corresponded to the deeper (> 200 m) water environments suggests that 3.4 Ga ago microbial communities of the photic zone were physically separated from the communities at the sea floor.

(iii) Archaeal metabolic pathways are very diverse; they include heterotrophy and several different types of chemotrophy [300], in particular, methanogenesis that is specific for Archaea [301]. This diversity suggests that members of the sea floor communities could have used different survival strategies and that their exodus from the deep-sea Zn-rich habitats may have proceeded in several waves. In particular, the strong Ni-dependence of enzymes that are involved in methanogenesis [301] suggests that this type of metabolism emerged within habitats that were particularly enriched in nickel, for example, in the form of NiS (millerite).

(iv) Cell membranes of Archaea are fundamentally different from that of Bacteria (see [187,302] and references therein). As argued elsewhere [73,303], this difference seems to indicate that the formation of modern ion-tight cell envelopes, needed to survive in chemically hostile environments, followed the separation of these two domains of life.

(v) Swimming motility mechanisms in different prokaryotic lineages are fundamentally different and evolutionarily unrelated [304]; this difference suggests that the LUCA could not swim. This deficiency, in turn, could have prevented a major gene exchange between the LUCA's descendants dwelling in different environments until the emergence of the first swimming apparata.

(vi) While Archaea contain many operational genes of supposedly bacterial origin, lateral gene transfer from Archaea to Bacteria was relatively minor and confined to hyper-thermophilic organisms, such as *Thermotoga maritima *[305,306]. This inequality of the lateral gene flows to and from Bacteria supports the straightforward possibility that the "horisontal" gene transfer between Bacteria and Archaea/Eukarya predominantly proceeded downwards relative to the Earth surface, and could be essentially driven by sedimentation processes.

(vii) No Eukarya-specific autotrophic mechanisms have been reported so far [64]; their absence might indicate that pro-eukaryotes relied on heterotrophy;

(viii) At least 90% of animal biomass of the modern hydrothermal vents depends on the chemoautotrophic endosymbionts (mostly sulfur- and hydrogen-oxidizing prokaryotes [104,307]). Even single-celled Protozoa at hydrothermal vents exploit prokaryotic symbionts [308]. These numerous symbioses might reflect a long-lasting cooperation between the larger heterotrophic inhabitants of the vent communities and the smaller prokaryotes capable of chemoautotrophy; such cooperation may have eventually driven the emergence of eukaryotes [293,296].

(ix) The fraction of Zn-containing enzymes in Eukarya is higher than in Bacteria or Archaea, see Table 5 and refs. [124,250]. This observation is consistent with the suggestion that pro-eukaryotes remained adherent to the ZnS settings for a longer time than the ancestors of other lineages.

**Table 5:** Distribution of zinc-, non-heme iron- and copper-binding proteins in the three domains of life

	**Zn^a^**	**Non-heme Fe^b^**	**Cu^c^**
Bacteria	4.9	3.9	0.3

Archaea	6.0	7.1	0.4

Eukarya	8.8	1.1	0.3

The numbers show the fractions (in %) of proteins with binding sites for Zn, non-heme Fe and Cu, respectively, as related to the total protein content, in the representatives of the three domains of life, determined by application of the same bioinformatics approaches [135].

a – Based on the data from [250];

b – Based on the data from [251];

c – Based on the data from [311];

Several authors who have noted this prevalence of the Zn-dependent enzymes in eukaryotes (see e.g. [124,250]) attributed it to the evolutionarily recent proliferation of Zn-binding motifs (in particular, zinc fingers) among the Eukarya. The abundance of the Zn-dependent enzymes in Eukarya [124,250,309,310], however, is likely to be an ancient feature because it is complemented by the relative deficiency in the Fe-containing enzymes. The Fe deficiency follows from the quantitative estimates (see Table 5 and [251,311]), as well as from functional considerations: eukaryotic cells use the mitochondrial assembly systems to insert FeS clusters in the apo-proteins of their cytoplasmically and nuclearly located enzymes [312]. In most cases, an apo-protein is translocated across two mitochondrial membranes into the mitochondrial matrix, the FeS cluster is assembled and inserted, and the folded protein is translocated back into the cytoplasm across the same two membranes; it is still unclear whether and how the internal mitochondrial membrane maintains electric potential of ca. 200 mV while a folded, FeS cluster-containing protein is being translocated across it. The absence of full-fledged cytoplasmic machinery for assembling FeS clusters in eukaryotes might have several explanations. It is possible that the pro-eukaryote possessed the respective enzymes but they were later replaced by the more efficient machinery of its α-proteobacterial endosymbiont. However, it is hard to imagine that the (hypothetical) pro-eukaryotic machinery could be even less efficient than the described, extremely complicated procedure of inserting FeS clusters into the cytoplasmic apo-proteins by the mitochondrial enzymes. In our opinion, it is more probable that the pro-eukaryote just could not deal with FeS clusters because it dwelled in Fe-deficient environments. This certain incompetency of the pro-eukaryote in dealing with Fe follows also from the fact that eukaryotes use the heme biosynthesis enzymes that are specific for α-proteobacteria and that, most likely, were acquired from the α-proteobacterial endosymbiont [313]. Therefore eukaryotes may have colonized Fe-rich habitats later than the representatives of other domains, i.e. only after a pro-eukaryote entered into a symbiosis with a respiring α-proteobacterium that provided the host with the Fe-processing enzymes. The emergence of respiring α-proteobacteria should, however, follow the oxygenation of the ocean, at 2.0–2.5 Ga [127]. If so, pro-eukaryotes may have thrived and evolved in Zn-rich settings for at least 1 Ga, between the separation of the main domains of life and the oxygenation of biosphere [296,314]. Thus, not only the LUCA likely dwelled in the Fe-deficient, Zn-rich settings (see above), pro-eukaryotes may have inhabited these environments as well, and for quite a long time.

## Outlook: Potentially promising directions of the future research

Based on the arguments from this paper and the accompanying article [97], we would like to submit that the Zn world hypothesis has successfully passed the first set of trials and therefore seems to be worth of further testing. We believe that a combination of the "bottom up" and "top down" approaches might be decisive for the further validation of the concept.

### Potential "bottom up" trials

The most straightforward "bottom up" trial is to simulate the events in the primordial Zn world. The primordial photosynthesis can be simulated by using porous precipitates of ZnS and MnS (nano)particles covered by a solution that contains phosphite and other relevant ions, is saturated with H_2_ and N_2_/NH_4 _^+  ^in reasonable concentrations, and is set under the CO_2 _pressure of more than 10 bar. After illumination of this mixture by strong UV light, organic compounds are expected to form at the photosynthesizing surfaces. Upon simulations, it seems reasonable to vary the parameters which might affect the yield of photosynthesis, in particular the relative amounts of MnS, Mg^2+^, K^+^, Na^+^, N_2_, phosphite, ammonium, and so on. The tricky task would be determining the exact chemical nature of the ZnS-adsorbed reaction products; fortunately, some relevant approaches have been recently developed [43,94], so that this task, hopefully, could be accomplished. The primordial (photo)chemistry at the surface of a photoactive semiconductor might dramatically differ from the textbook biochemistry that describes the interactions of chemically stable compounds. It, however, might be inferred from experimentation. The recent synthesis of activated pyrimidine ribonucleotides from cyanamide, cyanoacetylene, glycolaldehyde, glyceraldehyde and inorganic phosphate in a reaction that bypassed free ribose and the nucleobases serves as a remarkable example of such an approach [33].

Simulations of the Zn world could also start from other "entry points". Under the above described conditions, (photo)polymerization of *pre-formed *nucleotides (or nucleosides in the presence of phosphite) could be studied at the ZnS surfaces. The encouraging results that were obtained upon studying the (photo)polymerization reactions at TiO_2 _surfaces [94], suggest that nucleotide polymerization at illuminated ZnS surfaces could proceed with an acceptable quantum yield.

The interactions of pre-formed RNA polymers with ZnS/MnS nanoparticles (quantum dots, see the accompanying article [97]) and their aggregates also deserve investigation. So far, such studies were mostly focused on the interactions between DNA molecules and CdS nanoparticles (see e.g. [315-318] for reviews and the accompanying article [97] for further references). Studies of the interactions between RNA molecules and ZnS/MnS nanoparticles under simulated primeval conditions (see above) could shed light upon the earliest events in the RNA World. In particular, it seems worthy to check the influence of ZnS surfaces on the activity of ribozymes.

In addition, the stereoselectivity of the ZnS-mediated photocatalysis (see the accompanying article [97] and references therein) can be experimentally tested by applying the already existing approaches [319-323] to the substrates that might be relevant in the context of abiogenesis.

Since structures of key biological molecules and of ZnS/MnS nanoparticles are all known, it might be worthwhile to perform a computer modeling of the interactions between biopolymers at the ZnS (MnS) surfaces. The interactions between RNA strands, as well as associations of protein chains and RNA molecules could be modeled at the surface of ZnS templates; the results of such simulations might be of great interest for understanding the ZnS-mediated primeval syntheses.

### Potential "top-down" tasks

A formidable "top-down" task is to reconstruct the tentative biochemistry of the Zn world using bioinformatics approaches. Comparative genome analysis made it possible, by searching for the common genes in Bacteria, Archaea and Eukarya, to reconstruct the gene repertoire that was responsible for the translation and transcription in the LUCA [196,197]. However, this approach did not allow uncovering the metabolism of the LUCA because the metabolic enzymes, owing to the widespread lateral gene transfer [324], rarely could be definitely attributed to a particular lineage (as, for example, the F-type membrane ATPases to Bacteria and, respectively, the A/V-type membrane ATPases to Archaea and Eukarya [205]). The analysis presented here indicates that the initial steps of evolution proceeded in the habitats that were rich in Zn, but deficient in Fe. Hence, Fe-dependent enzymes were unlikely to be involved in primordial metabolism. With the data from Table 2 on the oldest protein folds, it seems feasible to reconstruct the initial biochemistry by identifying the metabolic pathways that (i) predominantly involve Zn- and Mn-dependent, but not Fe-dependent enzymes and (ii) use proteins with the oldest folds. Leslie Orgel, to whom we would like to dedicate this article, has noted in his brilliant, posthumously published work [9] that the Calvin cycle differs from the reverse citric acid cycle in its preferable usage of Mg and Zn as metal cofactors instead of Fe. Orgel wrote: "*It is interesting to compare the kind of chemistry involved in the Calvin cycle with that involved in the reverse citric acid cycle. In both cycles, almost all of the molecules involved carry two or more negative charges. In the Calvin cycle, the great majority of these charges are provided by phosphate groups, but in the reverse citric acid cycle, carboxylate groups are the only sources of negative charge. Furthermore, the only reduction that occurs in the Calvin cycle – the conversion of 3-phosphoglyceric acid to glyceraldehyde-3-phosphate – occurs via an acylphosphate intermediate. Reduction in the reverse citric acid cycle never involves a preliminary phosphorylation. Enzymes that use transition metal ions or iron-sulfur clusters play an important role in the reverse citric acid cycle, but are absent from the Calvin cycle, which uses Mg*^2+ ^*and occasionally Zn*^2+ ^*cofactors in its enzymes. It seems plausible, therefore, that the enzymes of the reverse citric acid pathway evolved in a region rich in transition metal ions and sulfur, whereas those of the Calvin cycle evolved where phosphate and magnesium were abundant. Presumably, one of these two cycles arose before the other. Is it possible to determine which came first by using information on biosynthetic pathways and genomics data? A decision on this question, though not directly relevant to the origin of life, would be of the greatest importance for understanding the history of protein-based metabolism on the early Earth" *(quoted from ref. [9]). In the framework of the Zn world concept, we can suggest that the Calvin cycle emerged first (see the previous section), while the citric acid cycle would arise later, concomitant with the Fe-containing electron-transfer (respiratory) chains. This suggestion agrees with the accepted fact that the amount of free phosphate in the biosphere has decreased with time (see [8,97,325] and references therein), so that metabolic cycles based on the phosphate usage, such as the Calvin cycle, should be evolutionarily older.

Last but not least, life continues to flourish within the ZnS-coated, deep sea hydrothermal fields, with their inhabitants categorized mostly as Archaea [115,117]. It might be worthwhile to inspect those ZnS-confined communities more closely. Although the ocean waters are saturated by oxygen, the interiors of the chimneys remain anoxic, because of the reduced state of hydrothermal fluids, so that many inhabitants of the vents are obligatory anaerobes [104]. There is a small chance that the descendants of pro-eukaryotes might still thrive in the anoxic, porous ZnS edifices.

## Conclusion

In this article we have validated the Zn world hypothesis by checking the predictions that followed from it. In addition, we have shown that this hypothesis explains several observations which so far remained without acceptable rationalization. Since the hypothesis has passed all these trials, its key suggestion, namely that the development of the first life forms could take place within the photosynthesizing ZnS edifices of hydrothermal origin, appears to have been validated. Further details of the primeval Zn world, and the exact physics and chemistry of the reactions involved, deserve further clarification.

In the course of this study, we have analyzed the available data on the relative abundance of transition metals in biological systems. We have found that RNA molecules and oldest protein folds are associated with Zn and Mn, but not with Fe. It seems likely therefore that the early evolution proceeded in several distinct steps, namely (i) the "Zinc Age" with the first replicating entities "grazing" within photosynthesizing ZnS compartments and evolving into the first proto-cells, (ii) the "Iron Age", during which the organisms, after cessation of the ZnS-mediated photosynthesis, adapted to using abundant, but redox-active iron atoms in their energy-converting devices, and (iii) the "Oxygen Age" when the increase in the atmospheric oxygen content has driven the major evolutionary changes aimed at prevention of the oxidative damage to aerobic organisms. The transitions between these "ages" probably represent major evolutionary bottlenecks.

While the transition from the anoxic to oxygenated biosphere has been long recognized as a key evolutionary event (see e.g. [295,297,326,327]), the transition from the Zinc Age to the Iron Age, as well as the very existence of the primeval Zn world remained unnoticed until now. It seems likely that the "Lost Zn World" has not been uncovered earlier because the spectroscopic elusiveness of Zn hindered the experimental studies of Zn-containing systems. As a result, the importance of Zn for cell biology has been by and large underestimated (as a notable exception we would like to acknowledge the contribution of Vallee and co-workers who focused on Zn-containing enzymes for several decades [248,328]). Only in recent years, Zn-dependent systems have drawn more attention and the fundamental role of Zn started to get recognized (see [124,245,246] and references therein). We hope that this article could contribute to a shift from the Fe-centric inorganic biochemistry to the Zn-centric one that would better reflect the key role of Zn in the living nature and its evolution.

The Zn world scenario, by implying that the Zn- and Mn-dependent enzymes preceded the Fe-dependent ones, offers a new tool – a biochemical time arrow – for the analyses of the earliest evolutionary events. Up till now, there was no clear way to arrange cellular systems in the order of their evolutionary emergence (with the notable exception of the few oxygen-dependent enzymes that have seemingly replaced the oxygen-independent ones after oxygenation of the atmosphere, see [327] and references therein). Although some features of the lost Zn world are reconstructed in this and the accompanying articles, a lot of further work would be needed to understand the earliest steps of life on the Earth.

Finally, this work suggests that origin of life was not a one-time historical accident but a natural and, perhaps, potentially inevitable consequence of an interplay between the solar UV-light and the geochemical conditions that existed once on the ancient Earth.

## Competing interests

The authors declare that they have no competing interests.

## Authors' contributions

Both authors analyzed the data, wrote and revised the paper.

## Reviewers' reports

### Reviewer 1

Arcady R. Mushegian, Stowers Institute for Medical Research, Kansas City, MO, USA

This is the second of two thought-provoking manuscripts that discuss the hypothesis of the "Zn world" in which Life on Earth may have emerged. This manuscript is largely devoted to devising the ways of testing the traces of the Zn world in the present-day world of RNA and proteins. The computational experiments outlined in the manuscript are, in my opinion, the preliminary thoughts, which are unfortunately not set in a robust quantitative framework. More specifically:

Prediction 1. The elevated Zn^2+ ^content of the primordial environments should be conserved inside modern cells

-- Please elaborate which content should be conserved – is it total concentration or free ion concentration? The latter is very low – is it because Zn^2+ ^ion is nowadays toxic and needs to be sequestered? When in evolution did it become so?

***Authors' response***. *In the revised version of the paper we have changed the order of predictions, this particular prediction has been moved to no. 5. We now explicitly indicate that it is the total concentration of Zn that should be conserved. Actually, zinc is relatively non-toxic, at least for humans who can tolerate it in fairly large amounts *[329]. *We believe that the low levels of free Zn in the cells reflect certain Zn limitation of modern organisms and, perhaps, justify the currently popular inclusion of Zn-containing complexes into the vitamin sets*.

### Reviewer 1

Prediction 2. There should be ribozymes with Zn-dependent catalytic activities.

-- I think that an honest conclusion would be that direct evidence of this is weak, though synthetic ribozymes that can use Zn^2+ ^ions are known.

***Authors' response***. *Generally, we agree with the referee's comment. In more detail we consider this topic in our response to Koonin*.

### Reviewer 1

Prediction 1. RNA structures should be enriched in Zn^2+ ^ions.

-- The way I understand the data, the presence of in the Zn^2+ ^ions in known RNA structures in a form directly bound to RNA is not much different from Mn and is much less common than Mg. Computing the enrichment statistics here and everywhere else in the study may help, though I appreciate the difficult issues that have to do with the sampling.

***Authors' response***. *In this work, to avoid a "self-serving" bias, we based on the data available either from the literature or from the publicly-accessible databases. We would like to emphasize that an accurate checking of each prediction is a task that would have required writing a separate article*.

### Reviewer 1

Prediction 3. Zn^2+ ^ions should be associated with the evolutionarily oldest protein folds;

-- This again should be a quantitative argument, but I am not sure what it actually is: associated with the oldest folds more than with the younger ones? Associated more than other divalent cations? More than what should be expected by chance? More specifically: the particular set of ubiquitous folds that are taken as a (most likely, reasonable) proxy for the oldest folds are clearly rich in divalent cation-binding proteins. Many families of dinucleotide-binding Rossmanoids, nucleotidyltransferases and polymerases of different classes – all of them use Mg^2+ ^in preference to everything else, and if there is no Mg^2+ ^in the crystal growth media, occasional Zn^2+ ^will substitute. In these classes, there is no such thing as "Zn or Mg".

***Authors' response***: *We agree that a more systematic study of metal occurrence in the "oldest" and "youngest" folds would be highly desirable. Here we just counted metal ions that are present in the crystal structures. Thereby we did not check whether Mg*^2+ ^*was present in the crystal growth media or not (usually it was). The fact that Zn*^2+ ^*ions were seen in many structures in spite of the presence of Mg*^2+ ^*ions in crystallization media might indicate that in certain cases (e.g. in zinc fingers) Mg*^2+ ^*ions could not substitute for Zn*^2+ ^*ions*.

### Reviewer 1

Prediction 4. Enzymes with evolutionarily "old" functions should depend on Zn^2+^;

-- I suggest either obtaining Zerkle data and examining them more closely, or not discussing them at all.

***Authors' response***: *We decided to retain the data of Zerkle and co-workers as unbiased evidence, but merged this prediction with the one on the function takeover from ribozymes by first enzymes. In the original version of the manuscript, while discussing the data of the data of Zerkle and co-workers, we have argued that they may have overestimated the fraction of iron-containing enzymes at the stage of the "very early life" (see also below for a related point made by Forterre). For example, we believe that iron-rich respiratory enzymes – assumed to be present from the very beginning by Zerkle and co-workers *[125] – *were not needed before the oxygenation of the atmosphere. In the revised manuscript we have dropped this discussion for the sake of brevity. These points deserve be considered in a separate publication*.

### Reviewer 1

Prediction 4a. The enzymes that emerged to take over the catalytic functions from ribozymes should be dependent on Zn^2+^

-- Why this would be the case? Transition from the RNA World to the Protein World may have occurred later than the Zn world, and other metals, like Mg^2+^, may have taken over the catalytic roles already. This seems to be better compatible with the evidence, does it not?

***Authors' response***: *In the revised manuscript we argue, in response also to the comments of Koonin, that the Last Universal Common Ancestor still dwelled in Zn-rich habitats. Then the transition from the RNA World to the RNA/Protein World would also have proceeded in Zn-enriched environments. The amount of Mg*^2+ ^*in the sea water is high and apparently always has been high. However, since Mg*^2+ ^*is a poor Lewis acid, certain catalytic tasks require transition metals. Accordingly, we argue that many catalytic activities that are common for enzymes and ribozymes are catalysed by zinc-dependent enzymes*.

### Reviewer 1

Manuscript as a whole: There is no discussion of the effect of various concentrations of the Zn^2+ ^ion on the stability of the phosphoester bond. There is related discussion of Fe^2+ ^ions on pg 17, but not of Zn^2+ ^ions.

***Authors' response:****The important topic of the Zn-catalyzed cleavage of phosphoester bonds (see also the comment by Forterre) is now included in the accompanying article *[97]* and is additionally discussed in the author's response to reviewers of that article*.

### Reviewer 1

pp. 26–31 are quite redundant with the first manuscript – consider shortening?

***Authors' response***: *The redundant parts have been streamlined*****.

### Reviewer 1

p. 34 – items (iv) and (v) sound very reasonable to me, but I do not see how they relate to Zn world. The same for items (vii) and (viii) on p. 35.

*Authors' response:*

*Item (iv): The fundamental difference between the membranes of Bacteria and Archaea indicates that modern membranes developed separately in the two domains. Then the Last Universal Common Ancestor (LUCA) must have had primitive (if any) membranes that could not be particularly ion-tight and should have enabled ionic equilibration with surrounding media *[73,138,187,206]. *In the revised manuscript, we argue that the high Zn content of modern cells could be traced to the ionic equilibrium between the interior of the LUCA and its Zn-rich environment*.

*Item (v): We suggest that the separation of the major domains was driven by the drift of the ZnS-confined seafloor communities away from the continental phototrophic ones. If the LUCA had swimming gadgets, these communities could continuously mix and exchange genes, preventing the development of Bacteria- and Archaea-specific traits*.

*Items (vii) and (viii): Item (viii) became (ix) in the revised manuscript. The absence of eukaryote-specific, iron-processing machinery even in modern Eukarya strongly indicates that pro-eukarya could not rely upon iron-dependent redox metabolism. The only metabolic alternative would have been heterotrophy of some kind relying on the Zn-dependent hydrolases*.

Reviewer's response in a second review

The distance that a swimming LUCA can cover: how does it compare with the velocity of continental drift?

***Authors' response***: *The continental drift was unlikely to take place in Hadean. It is believed that at that time, cooling of the Earth, the main cause of contemporary continental drift, was mediated by numerous "hot spots" resembling modern Iceland *[49]. *However, the importance of swimming for the gene exchange between the major domains could be evaluated by estimating the rate of "sinking" of the Zn-rich habitats. Using estimates of the atmospheric pressure of ca. 100 bar after condensation of the ocean at 4.4 Ga *[52,53]*and 2–6 bar at 3.3 Ga *[50]*and assuming a linear decrease of pressure with time, it is possible to calculate that the pressure may have decreased, on average, by 1 bar in ~10 million years. When the pressure falls below certain threshold value (ca. 10 bar), the highest temperature of hydrothermal fluids drops below ~200°C and they become depleted of Zn. Therefore, after the atmospheric CO*_2 _*pressure dropped below 10 bar, the Zn-rich hot springs could function only at a certain depth, where the total pressure – of the atmosphere and the water column – remained above 10 bar. A 10-meter water column produces pressure of ca. 1 bar. Therefore the submersion of the Zn-rich hydrothermal systems should have proceeded with a rate of ca. 10 meters in 10 million years, or ca. 1 micron per year, slow enough to be overcome by any kind of swimming motility. Therefore, if LUCA and its immediate descendants could swim, the phototrophic, swimming organisms could occupy the surface water layer (the photic zone) just above the sea-floor communities. The sedimentation of these organisms would promote sharing of genes and would have prevented crystallization of the domain-specific traits. In contrast, non-swimming organisms would have stayed confined to their habitats, so that the sub-aerial phototrophic communities could not, at least initially, exchange genes with the see-floor, ZnS-confined communities*.

### Reviewer 1

p. 36 line 19: I do not see why these explanations are parsimonious or why this would necessarily be a good thing.

***Authors' response:****Parsimony is a good thing. If one finds an explanation that simultaneously covers several unresolved items, the probability that this hypothesis captures some actual features of a natural phenomenon is higher than when one has to come up with a separate explanation for each unsolved item*.

**Reviewer's response in a second review: **You explained why you think parsimony is good but not why you think these explanations are parsimonious.

***Authors' response***: *The Zn world concept may be considered a parsimonious explanation of the three items listed in section on testing the explanatory power of the Zn world concept. because it explains them all. Alternatively, one could suggest separate explanation(s) for each item. For example, the prevalence of Zn in modern enzymes (Zn paradox) might be explained by the emergence of life within Zn-rich hydrothermal settings of sea floor. Then, however, the unique photochemical traits of nucleic acids would still remain unexplained. These unique traits, separately, were suggested to reflect the emergence of life in some illuminated settings *[78,119,120]. *If we now try to find a parsimonious explanation for the Zn paradox and the unique photochemistry of nucleic acids, the seemingly unique solution is the emergence of first life forms in some illuminated, Zn-rich settings. However, Zn could be present on primeval Earth only as ZnS, which, when illuminated, can reduce CO*_2_. *Hence a parsimonious explanation just for two items "bore" a solution to the problem of abiogenic CO*_2 _*reduction as well*.

### Reviewer 2

Eugene V. Koonin, National Center for Biotechnology Information, National Library of Medicine, National Institutes of Health, Bethesda, MD, USA

This paper strives to test the hypothesis that is put forward in the accompanying paper by Mulkidjanian, namely that life originated in a photosynthetic "Zn world" where Zn sulfide catalyzed a variety of synthetic reaction fuelled by the energy of UV radiation [97]. The idea of the pivotal role of Zn ions (and ZnS in particular) in the earliest stages of the evolution of life is highly attractive and generally plausible. However, in this manuscript, Mulkidjanian and Galperin put the plank very high by formulating several predictions that they claim to serve as Popperian tests of the "Zn world" hypothesis. In principle, the intention to test the hypothesis in a formal Popperian setting is indeed commendable. In practice, it is well known that it is hard to strictly adhere to Popperian criteria, and this paper is no exception. Unfortunately, as I see it, all the formulated predictions are weak, not unique to the "Zn World" hypothesis, and the nature of the evidence cited as being compatible with the predictions is such that one is prompted to ask "so what?" on most occasions. I briefly address the predictions and their purported tests one by one (the numbering of the predictions is mine – I think the authors should try to be more consistent to facilitate reading).

***Authors' response***:

*In relation to the popular view on the importance of iron for the primeval metabolism, our article gives a following answer to the "so what?" question: "the early life, most likely, developed in Zn-rich settings, its emergence in Fe-rich settings is unlikely. Free Zn*^2+ ^*ions, however, because of their poor solubility in primordial seawater, could have become available only if some process led to their continuous release – e.g. primordial abiogenic photosynthesis, as we suggest"*.

*The rationale beyond our decision to formulate a set of testable predictions was simple (see also ref*. [25]): *if several predictions prove to be correct, then the tested hypothesis could be fairly countered only by an alternative hypothesis that would explain all those predicted observations. Hence the number of different predictions has a value of its own*.

*The following paragraphs respond to the specific comments of the referee that are related to particular predictions; the more general comment is addressed at the end of our response*.

### Reviewer 2

1. High content of Zn in modern cells – predicted on the basis of the "chemical conservation" principle. The principle is extremely general and might not be of huge heuristic value but suppose we accept it. The problem with this prediction is that it is not specific at all. Yes, of course, there are many Zn-dependent enzymes in all cells, and Zn is concentrated compared to any environment. Is it relatively more abundant than other divalent cations? There is no answer in the paper. But, even if it was, does this link Zn to primordial stages of evolution? Or, if it was not, would that falsify the Zn world hypothesis? I doubt that either of these propositions holds. The prediction just is not specific enough.

***Authors' response***:

*1) In the revised manuscript we state explicitly that zinc is indeed relatively more abundant in cells than other divalent transition metals – if compared to the chemical composition of sea water – *see Table 1* in the accompanying article*.

*2) We added a new section devoted to the metallome of the Last Universal Common Ancestor. where we link the content of Zn in modern cells to primordial stages of evolution*.

*3) If this particular prediction would not prove to be correct, the hypothesis would not be falsified yet, but strongly weakened; the final outcome would then depend on the tests of other predictions. If they all would fail, the hypothesis would be falsified indeed. Fortunately for us, this is not the case*.

### Reviewer 2

2. There should be ribozymes with Zn-dependent activity. Provided the RNA world hypothesis is accepted, there could be a more serious prediction here. However, I think the authors are too permissive in their formulation. A strong prediction would be that ribozymes are, mostly, Zn-dependent or that certain classes of ribozymes that are most relevant to the origin of cells, such as polymerases and ligases, should be Zn-dependent. The way it stands, the prediction is too vague, whereas the data are very uncertain as the authors admit, even as they check this prediction in their favor.

***Authors' response***: *We admit that the test of this prediction has led to the least conclusive results. This has several reasons. (a) The metal specificity of ribozymes is low, such that Mg*^2+ ^*ions can occupy almost any divalent metal binding site; (b) the incubation media that are used by RNA scientists usually contain no other divalent cations besides the large amounts of Mg*^2+ ^*(G. Yusupova, personal communication); and (c) there are only few types of natural ribozymes discovered so far, although, as we discuss in the manuscript, some their representatives show Zn-specific activity*.

*Among the native ribozymes, only the type I and type II introns can operate as ligases. Their activity requires divalent metals, but seems to be Mg-specific *[330]. *Generally, the activity of the known natural ribozymes is limited to the cleavage and formation of phosphoester bonds. The enzymes with this kind of activity are also Mg-dependent*.

*There are no natural ribozymes with polymerase activity. The artificially selected ones were shown to be Mg-dependent – since they were selected in a medium that contained Mg*^2+ ^*as the only divalent cation *[331]. *We are not aware about attempts to select ribozyme with a polymerase activity by using mixed Zn/Mg media. Such an approach proved to be successful when a ribozyme for aldol reaction – which is chemically more demanding than polymerization of nucleotides – was selected. In a selection medium that contained 5 mM MgCl*_2 _*and 0.3 mM ZnSO*_4 _*a Zn-dependent ribozyme was readily obtained *[176]. *The Zn*^2+ ^*ions were added to the selection medium since protein aldolases are Zn-dependent. As long as protein polymerases are Zn-dependent as well, we dare to predict that a selection of a Zn-dependent ribozyme polymerase is just a matter of a worthwhile trial*. *Generally, the hypothetical ribozymes of the RNA World should have been able to catalyze various chemical transformations; the example of enzymes shows that Zn*^2+ ^*ions may have been involved as catalysts in many of them*.

### Reviewer 2

RNA structures should be enriched in Zn compared to other transition metals. Again, this might make sense in the context of the RNA World. However, there are few RNA structures containing any transitional metals including Zn. The authors present a variety of post hoc considerations to explain the paucity of these ions – all this might be true but as a result, the argument does not seem to be convincing. An interesting observation in this section is the absence of Fe in any RNA structures, and the authors' interpretation that iron is likely to catalyze RNA cleavage makes sense. However, the data that are cited in support of this idea, on ribozyme activation by iron (ref. 179), logically suggest the opposite of their argument, namely, that iron is tolerated by RNA molecules, at least molecules like ribozymes that seem to be most relevant for the RNA World.

***Authors' response***: *This is a very important point, so we have revisited the experimental protocols in ref*. [332]* (no. 179 in the original manuscript). In these experiments, it was checked whether diverse metals could activate a particular ribozyme, which was pre-selected for the ability to be activated by Mn*^2+^, *Co*^2+^, *Ni*^2+^, *Zn*^2+ ^*and Cd*^2+^. *Thirty eight diverse metal chlorides were added at 100 μM to the samples that contained precursor RNA and a ribozyme, the reaction was terminated after 5 min incubation at 23°C by the addition of a buffer supplemented with EDTA to the final concentration of 40 mM. The reaction products were then electrophoretically separated and the extent of the precursor RNA cleavage was checked. Of the 38 metals tested, only Fe*^2+ ^*ions were able to induce cleavage of the precursor RNA. However, this procedure seems to be inappropriate for Fe*^2+ ^*ions. In such a set-up there is no way to discriminate whether the precursor RNA had been cleaved by the Fe*^2+^*-activated ribozyme during the 5 min incubation or by the Fe*^2+^*-EDTA complexes after the anticipated reaction termination. In fact, Fe*^2+^*-EDTA complexes are widely used as cleavage agents for RNA *[168]. *Because of this uncertainty, we have decided to drop the discussion of the work of Zivartis et al*. [332].

### Reviewer 2

3. Zn ions should be present in oldest protein folds. I will refrain from the criticism of the work of Bourne's group – let us assume that the inference of the oldest folds there is reasonable. Zn is found in a rather small minority of representative structures, albeit from many folds. This is hardly surprising given that, oldest or not, these are indeed very common folds. Perhaps, a comparison with "less ancient" folds would help (it is unclear why such a comparison is not included) but then, again, I am rather skeptical as there can be many reasons why some protein structures contain a particular metal whereas others do not. There is just not bridge from here to the "Zn World".

***Authors' response***: *We agree that checking of the metal content of the "less ancient" folds is a worthwhile task. This task, however, is beyond our current capabilities since the total number of fold superfamilies is about 1300. We, however, would be happy if somebody carried out this work. As already noted, we have added a new section on the metallome of the LUCA where we tried to build a bridge from the traits of modern proteins to the primeval Zn world*.

### Reviewer 2

4. Occurrence of Zn in enzymes with the oldest functions. I find this line of argument more interesting than the preceding 4 lines. It is therefore somewhat unfortunate that the authors do not perform any analysis of their own but rather limit themselves to the citation of [125] where the exact list of "primordial enzymes" is not given. This lack of concreteness seriously weakens this potentially relevant argument.

***Authors' response***: *In this work we deliberately relied on the data sets obtained by others, to avoid a potential bias. Whatever mistakes could have been made by the authors of those data sets, neither of them had any intention to provide supporting evidence for the Zn world concept*.

### Reviewer 2

5. Presence of Zn ions in enzymes that could have taken over ribozyme activities. The argument here is long and convoluted, and I am afraid I cannot conclude that there is any strong data presented.

***Authors' response***: We *shortened this section and merged it with the section on the Zn-dependence of evolutionarily old functions*.

### Reviewer 2

In the beginning of the next section, it is claimed that the Zn World hypothesis "has successfully passed all six falsification tests". I am afraid that I cannot condone this statement. To me, there is only one serious falsification test here, #4. Indeed, if there were no Zn-dependent enzymes among the ones assumed to be primordial, that could be construed to falsify the Zn World hypothesis. Unfortunately, as noticed above, the analysis of this prediction in the paper is not the strongest. The rest of the predictions, to me, either do not follow from the Zn World hypothesis or are too weak and vague – self-serving, within the framework of the Popperian paradigm – to provide any argument in support of the hypothesis (this is not quite Popperian language but, at the end of the day, when a hypothesis passes several falsification tests, this result constitutes support, and I think this is how the authors see the situation).

***Authors' response***: *In response to reviewers' comments, we have added a new section on the metallome of the LUCA, which discusses the link between the relatively high Zn levels in modern cells, enzymes, and RNA structures and the primordial stages of evolution. We argue that the Zn enrichment of modern cells/RNA structures/enzymes is evolutionarily relevant and supports the Zn world hypothesis. In addition, we now analyze the metal content of the proteins that supposedly were present in the LUCA. To minimize the authors' bias, we have used the data set that was obtained by the Reviewer himself *[196]. *We show that proteins that supposedly were present in the LUCA predominantly contain Mg*^2+ ^*and two transition metals, Zn and Mn*.

*Our predictions are not "self serving" in the Popperian sense – to our knowledge he has never used such a term in relation to the falsification tests. The tests can be either tautological or not – we cared to make predictions that are not related to the premises on which the hypothesis is built*.

*Our predictions, however, are obviously "self-serving" in the common sense of word – we try to gather support for own hypothesis. Of course, we would be happy to see this hypothesis scrutinized by others*.

### Reviewer 2

I must note that I am generally sympathetic with the Zn World hypothesis and agree with the authors that substituting ZnS for FeS in the model of early evolution within networks of inorganic compartments is a promising idea. Moreover, I think the paper does include an argument that is not only compatible with this hypothesis but can be considered supportive. This is the so-called "Zn paradox" discussed in section 2.4.3. Indeed, properties of transition metals do not seem to explain why Zn is a cofactor for a disproportional number of diverse enzymes, and this is a provocative observation. Indeed, if a biological pattern does not have a functional or mechanistic explanation, one starts suspecting that it could be explained by the legacy of early stages of evolution – in this case, the Zn World. Perhaps, this argument could be addressed in somewhat greater detail.

***Authors' response***: *The respective section has been expanded. We additionally note that while prevalence of Zn in certain types of enzymes could be attributed to the catalytic properties of Zn*^2+ ^*ions, their ubiquitous involvement as structural elements *[128,210,243]* had no known explanation until now. The Zn world concept explains the paradoxical prevalence of Zn ions both as catalysts and as structural elements by the shaping – and folding – of first proteins in Zn-rich habitats*.

### Reviewer 2

I am rather frustrated with the discussion of the purported implications of the Zn World hypothesis for the divergence of the domains of life and subsequent evolution. I am not inclined to discuss these in much detail but I think the majority are far-fetched and some just do not seem to be serious like conclusions based on the higher content of Zn-dependent enzymes in eukaryotes compared to archaea and bacteria or the conclusions on symbiosis etc. [(ix) and (vii) in section 2.4.5, respectively] etc. In my opinion, it is highly desirable to simply drop this section.

***Authors' response***: *We have decided to retain some of this discussion; the respective part of the article has been, however, shortened and rewritten for the sake of clarity*.

### Reviewer 2

To summarize, I think the accompanying paper by Mulkidjanian together with this paper present a new, interesting, chemically plausible and overall promising hypothesis on the settings of primordial evolution. However, I think the authors do a disservice to their own good idea by heavily overloading the hypothesis with (at best) tangentially relevant arguments that they counter-productively attempt to present in a formal Popperian setting (I suppose, at least, in part, this approach is borrowed from Wächtershäuser but here it is taken to much greater and entirely unnecessary lengths). The paper is also full of implications that do not seem to really follow from the hypothesis but are discussed at great lengths. I would note that this paper (and I think the accompanying one as well) are heavily "overspecified", to the extent that there is a danger to completely drawn an interesting idea in a muddy waters of weak argumentation and excessive discussion. In my opinion, both papers could be reduced to a single, perhaps, 10 pages long article on the hypothesis that would discuss the relevant photochemistry and photophysics along with the interesting "Zn paradox". In that form, it would be a valuable contribution to the origin of life literature.

***Authors' response***: *Regular scientific papers are usually addressed to a handful of experts in a narrow research field. The potential audience of a paper on the origin of a life might be much broader and could include readers with different backgrounds, from mathematics to geology. Accordingly, their understanding of which arguments are convincing and which are only tangentially relevant might differ as well. In the view of this broad and unevenly trained potential audience, we have chosen a presentation style that, while being more diffuse as compared to routine scientific papers, might reach more readers*.

### Reviewer 2

A specific and relatively minor comment on horizontal gene transfer:

"The rigorous vertical inheritance of the genes responsible for information processing, in particular of the ribosome machinery [200], might indicate that these genes, at least at the LUCA stage, already formed the non-shared genetic cores of the first organisms"

The vertical inheritance of ribosomal protein genes let alone other components of information processing systems is by no account "rigorous", horizontal transfer of these genes is common enough [201,202] even if less common than horizontal transfer of genes for metabolic enzymes, although even that trend has been questioned [203].

***Authors' response***: *These references are now cited*.

### Reviewer 3

Patrick Forterre, Institut Pasteur, Département de Microbiologie, Unité de Biologie Moléculaire du Gène chez les Extrêmophiles, Paris, and Université Paris-Sud, Institut de Génétique et Microbiologie, Orsay, France

The origin of life remains a major unsolved problem in science, and no consensus exists in the community of people who try to tackle this problem, despite the great number of theories that have already been proposed. In an accompanying paper, Mulkidjanian proposed a new theory in which the first organisms were photosynthetizers, that used zinc sulfide (ZnS) formed in sub-aerial setting to capture solar energy for abiotic reduction of CO2. In this paper, Mulkidjanian and Galperin look in modern cells for support of this theory. By extensively reviewing the literature, they made six observations that could be viewed as supporting the idea that Zinc has indeed played a major role in early life evolution. In particular, they conclude from their survey of the experimental literature that ribozymes can work using Zinc as cofactor instead of magnesium, and that zinc is enriched in proteins with "old folds" or "old functions". This part of the paper is very interesting and can push the biochemists to look more carefully at the real metal dependency of the protein they are studying. These observations convince me that somehow Zinc should have played an important role in early biological evolution. An interesting observation is that the fraction of Zn-containing enzymes is higher in Eukarya than in Bacteria or Archaea. If the hypothesis of the author is correct, this supports heretical ideas according to which the proto-eukaryotic lineage might be more ancient than streamlined lineages of Archaea and Bacteria [333]. The authors call the ancestor of Archaea and Eukarya, a *proto-archaeon *or a *pro-eukaryotic archaeon*. This is based on the idea that evolution always goes from simple to complex and that streamlining in the archaeal domain has been much less important than complexification in the eukaryotic domain, something not so clear at the molecular level. Considering that Archaea are probably monophyletic [288], I think that the authors should avoid these confusing terms (proto-archaeon or a pro-eukaryotic archaeon) and only refer to the *pro-eukaryotic *ancestor.

***Authors' response***: *Throughout the manuscript, the pro-eukaryotic archaeon has been renamed pro-eukaryote*.

### Reviewer 3

More importantly considering the objective of this paper, I have a fundamental problem with the idea of an early Zinc world. Indeed, from my own lab experience, it is clear that Zinc has the ability to strongly induce cleavage of DNA, especially at high temperature [334]. Zinc has even a stronger deleterious effect on RNA. The cleavage of polyribonucleotide by Zinc has been extensively studies by Butzow and Eichhorn in the last century. These authors have shown in particular that RNA is especially prone to Zinc-induced degradation and even argued that the higher resistance of DNA to metal-induce degradation explains why DNA replaced RNA as cellular genetic material in the course of evolution [335]. The high sensitivity of RNA to Zinc should have created a great problem for early RNA-based systems (before the invention of proteins that could protect RNA against Zinc-induced hydrolysis). In any case, if the Zinc world really occurred it should have done so at low temperature. The Zinc world hypothesis therefore strongly supports the idea of a cold origin of life. It raises the possibility that life could have only invades high temperature environment after the emergence of DNA and modern complex proteins. In any case, this paper is important because it should stimulate investigators to resume the study the effect of Zinc on RNA stability in the presence of minerals, lipids and/or peptides, and to analyze the activity of various ribozymes in the presence of this metal at different temperatures.

***Authors' response***: *The possibility of the Zn-catalyzed cleavage, which was also addressed in the comment by Mushegian, is indeed a very important topic. We now consider this point in detail in the accompanying paper *[97]* and in the response to the Mushegian's comment to that paper. We argue that the ability of proteins to bind to the 2'-OH group of ribose could protect RNA molecules from Zn-catalyzed cleavage and may have driven the emergence of the first proteins. We are grateful to the Reviewer for pointing out the very useful reference to the work by Butzow and Eichhorn*.

### Reviewer 3

Although I found this paper really interesting, I think that the authors were misguided in connecting their Zinc world hypothesis to the Martin and co-workers hydrothermal vent scenario for the origin of life, in which, as described by Mulkidjanian and Galperin: "*Bacteria and Archaea are descendents of two distinct populations that thrive around hydrothermal vents*". The plural of vents in that description is misleading. Indeed, in that scenario, life should have originated and evolved up to modern cells in a single chimney, since life forms were trapped in mineral cages until the advent of Bacteria and Archaea. If the conditions were favourable to the development of a Zinc-world in cold hydrothermal settings, many independent primitive Zinc worlds should have emerged more or less simultaneously at many places at the earth surface. In the one chimney scenario, this means that all hydrothermal vents at the earth surface should have produce living organisms, but that Archaea and Bacteria originated from the same chimney (out of millions) and wiped out all other cells that were produced by other chimneys!! This seems ridiculous. Even if all ancestral chimneys were connected into a single giant chimney all around the globe, living organisms could not have moved and competed from one part of this giant chimney to another since they were trapped in mineralized cages (ZnS compartments), so LUCA and its descendents should have originated from the same region of this giant chimney, against wiping out cells that were produced by other parts of this chimney. In general, I don't think that one can trace the origin of the three domains to the evolution of the vent systems and I don't buy the geochemical scenario proposed by the authors for the origin of the three cellular domains. To explain the formation of the three domains, one should understand why three types of molecular biology (for instance three versions of the ribosomes) originated from LUCA, and this cannot be related simply to considerations based on various metabolisms.

***Authors' response***: *The expression "around hydrothermal vents" has been replaced by "within a deep sea hydrothermal vent"*.

*In contrast to Martin and co-workers, we think about the first biotopes as rings of precipitated ZnS/MnS particles around chimneys of continental hot springs. Based on the typical structure of ancient volcanogenic massive sulfide deposits *[261], *we can envision that these rings intersected and formed a continuous net of photosynthesizing and inhabited settings, most likely occupying the bottom lands of primeval hydrothermal fields. The life in these biotopes was confined to the illuminated ZnS surface, so the first life forms could be moved within these biotopes by water. Moreover, the illuminated ZnS compartments should have been fragile and break continuously because of photocorrosion*.

*Further, we speculate that upon the separation of the main lineages not the organisms themselves, but the geologic settings that they inhabited started to move away from each other. With the decrease in the CO*_2 _*atmospheric pressure, inhabited ZnS-rich settings at hydrothermal vents would move deeper and deeper into the ocean, away from the continental phototrophic communities. As we believe, exactly the initial lack of swimming motility of the first organisms enabled their separate evolution and crystallization of the domain-specific features in the phototrophic communities (future Bacteria), on the one hand, and chemotrophic/heterotrophic communities on the sea floor (future Archaea/Eukarya), on the other hand*.

### Reviewer 3

Generally speaking I think that the authors, as many other scientists working in the origin of life fields, tend to underestimate the various evolutionary steps that were required to go from the first proto-cells to LUCA and later on to modern cells (Archaea, Bacteria and Eucarya). This is clear when the authors speak of proteins that have been "*attributed to the very early life*", including in that category DNA repair and replication proteins. For me these proteins do not testify for the very early life because they could have only appeared after the evolution of very sophisticated ribosomes, at a very late period of early life evolution. If the first life forms indeed originated in ZnS compartments of an hydrothermal vent, I would suggest that the cells that emerged from these vents were primitive cells that started to compete with each others as free living cells in ancestral water ponds and/or in shallow waters of the ancestral oceans. They might have been pre-RNA cells or cells that found a way to protect their RNA against Zinc-induced degradation. This does not dismiss the possibility that the first proteins that replaced ribozymes after the invention of ribosomes mainly inherited zinc ions from the catalytic centers of these ribozymes, or that cells of that time (second age of the RNA world) were still thriving in a rich zinc world.

***Authors' response***. *We agree that attribution of DNA repair and replication proteins to the very early life, as done by Zerkle et al*. [125], *is controversial. In the revised manuscript we still present their data but do not discuss them (see also our response to the respective comment of Mushegian). We fully agree with the Reviewer that acquisition of Zn*^2+ ^*ions may have preceded the emergence of DNA repair proteins. In the revised version we added a new section on the metallome of the LUCA, where we argue that the first organisms could have thrived in the Zn-rich environments up to the stage of the LUCA*.
